# Effectiveness and implementation of psychological interventions for depression in people with non-communicable diseases in South Asia: Systematic review and meta-analysis

**DOI:** 10.1080/00207411.2023.2202431

**Published:** 2023-04-24

**Authors:** Gerardo A. Zavala, Hannah Maria Jennings, Saima Afaq, Ashraful Alam, Naveed Ahmed, Faiza Aslam, Aatik Arsh, Karen Coales, David Ekers, Mumtahana Nabi, Anum Naz, Nayeema Shakur, Najma Siddiqi, Judy M. Wright, Ian Kellar

**Affiliations:** a Department of Health Sciences, University of York, York, United Kingdom; b Hull York Medical School, York, United Kingdom; c Institute of Public Health and Social Sciences, Khyber Medical University, Peshawar, Pakistan; d Diabetic Association of Bangladesh, Dhaka, Bangladesh; e Institute of Psychiatry (IoP), Benazir Bhutto Hospital, Rawalpindi Medical University, Rawalpindi, Pakistan; f Tees Esk and Wear Valleys NHS FT, York, United Kingdom; g ARK Foundation, Dhaka, Bangladesh; h School of Medicine, University of Leeds, Leeds, United Kingdom; i School of Psychology, University of Leeds, Leeds, United Kingdom

**Keywords:** Systematic review, depression, non-communicable diseases, South Asia, policy, psychological interventions

## Abstract

We evaluate the effectiveness of psychological interventions for depression in people with NCDs in South Asia and explore the individual, organizational, and policy-level barriers and facilitators for the implementation and scaling up of these interventions. Eight databases (and local web pages) were searched in May 2022. We conducted random effects models to evaluate the pooled effect of psychological interventions on depression in people with NCDs. We extracted the individual, organizational, and policy level barriers and facilitators. We found five randomized control trials, nine qualitative studies, and 35 policy documents that fitted the inclusion criteria. The pooled standardized mean difference in depression comparing psychological interventions with usual care was −2.31 (95% CI, −4.16 to −0.45; *p* = .015, *I*^2^ = 96.0%). We found barriers and facilitators to intervention delivery, mental health appears in the policy agenda in Bangladesh and Pakistan. However, there is a lack of policies relating to training in mental health for NCD health providers and a lack of integration of mental health care with NCD care. All of the psychological interventions reported to be effective in treating depression in this population. There are important delivery and policy barriers to the implementation and scaling up of psychological interventions for people with NCDs.

## Introduction

People with physical non-communicable diseases (NCDs) have a two to three times increased risk of depression (Lloyd et al., 2018; Mendenhall et al., 2014). Depression and NCDs have a bidirectional relationship with shared biological and environmental determinants (Singer et al., 2017); each condition adversely affects outcomes for the other. Depression alongside NCDs is associated with a 1.5 higher risk of mortality (Van Dooren et al., 2013), impaired self-management, increased NCD complications, and longer duration and recurrence of depressive episodes (Egede & Ellis, 2010; Lustman et al., 2000). Depression significantly increases healthcare costs for NCDs, contributing to the loss of earnings and employment, stigma, and poorer quality of life (Holt et al., 2014; Lloyd et al., 2012; Mommersteeg et al., 2013; Sartorius, 2018). Depression and NCD multimorbidity significantly contribute to global health and socio-economic inequalities; the prevalence of both is increasing faster in low and middle-income countries (LMICs) than in high-income countries (HICs) (Mendenhall et al., 2014).

Depression is treatable with relatively low-cost psychological and/or pharmacological therapies (WHO, 2014), which are relatively simple and potentially culturally portable (Hendriks et al., 2018). Psychological interventions have been shown to be effective in treating depression in people with NCDs in HICs (Uphoff et al., 2020). However, there is scarce information on LMICs where limited resources, income constraints, and differing socio-cultural acceptability are additional barriers to delivering these interventions (Dawson et al., 2015).

To the best of our knowledge, there are no systematic reviews addressing the effectiveness and barriers, and facilitators of psychological interventions for depression in people with NCDs in LMICs. Such trial-based evidence is essential to inform practice and the development of tailored interventions for LMIC, where information from high-income countries may not necessarily be applicable (Uphoff et al., 2020).

This review evaluates the evidence for psychological interventions for treating and managing depression (including their implementation) in people with non-communicable diseases in South Asia. For the policy analysis, we will have a particular focus on two countries, Bangladesh and Pakistan, where both depression and NCDs are rapidly increasing and where we aim to test the acceptability and effectiveness of a psychological intervention for depression in people with diabetes. According to the 2018 WHO progress report, an average person in Bangladesh and Pakistan between the ages of 30–70 carries a 21–25% risk of premature death from NCDs (WHO, 2018), and the prevalence of depressive disorders range from 15.3 to 56.6% among people living with NCDs in these countries (Hossain et al., 2020).

## Aims

The aims of this study are (1) to evaluate the effectiveness of psychological interventions for the treatment of depression in people with NCDs in South Asia; (2) to explore the individual, organizational, and policy-level barriers and facilitators for implementation and scaling up of these interventions.

## Methods

We conducted the review in line with Center for Reviews and Dissemination guidance on the conduct and reporting of systematic reviews (Akers, 2009). and with the Preferred Reporting Items for Systematic Review and Meta-Analyses (PRISMA) guidelines (Page et al., 2021).

### Data sources and search strategy

All the searches were conducted on October 2021 and updated on the second week of May 2022; ASSIA (ProQuest), CINAHL (EBSCOhost), Embase Classic + Embase (Ovid) 1947–2022 May 4, Global Health (Ovid) 1910–2022 Week 17, IMEMR (WHO Global Health Index Medicus), ISMEAR (WHO Global Health Index Medicus), Ovid MEDLINE(R) ALL 1946 to May 4, 2022, and APA PsycInfo (Ovid) 1806 to April Week 4 2022.

Two searches were conducted in these databases to identify studies of BA for people with NCDs and depression in South Asia. Search 1 used search terms for the concepts: behavioral activation, psychological interventions, NCDs, depression, and South Asia. Search 2 attempted to ensure an exhaustive search for potentially relevant studies from Bangladesh and Pakistan by searching for all mental disorder terms (instead of just depression) and limiting them to Bangladesh and Pakistan (rather than all of South Asia).

The searches were developed iteratively, starting with previously used search strategies for behavioral activation and NCDs (Uphoff et al., 2020), psychological interventions (Kunzler et al., 2020), mental disorders (Mishu et al., 2021), and refining strategies iteratively to address the two search questions. Subject headings and free text words were used in each search concept; no limits were applied for language or publication date. Searches were designed by the Information Specialist and agreed upon with project team members.

The database searches were peer-reviewed by a second Information Specialist using the PRESS checklist (McGowan et al., 2016). Full line-by-line search strategies are available in the supplementary material. Further relevant studies were sought by citation searching (forwards and backward) of the included studies. The results of the database and citation tracking reference searches were stored and de-duplicated in an EndNote library.

For the policy documents, a third search strategy was developed only in Bangladesh and Pakistan. The inclusion criteria were: policy documents (legislation, strategy, and policy) related to mental health and/or NCDs in Bangladesh and Pakistan that are up to date; Articles (commentaries, research, etc.) to current legislation identified during the review, or internally, within the last 5 years. In addition to previously identified policy documents (through team members and contacts), we searched: (1) WHO Iris and World Bank Open knowledge repository, (2) In-country searches of government databases, and (3) In-country targeted Google search (See Table 1 in the Supplementary Appendix for full search strategy).

**Table 1.** Studies included in the effectiveness analysis.

Author, country, year	Setting	Sample size	Design	Outcomes	Comparator	Type of psychotherapy	Number of sessions; duration	Type of therapist	Delivery method	Women (%)	NCD
Ali et al. (2020) India 2020	Out patients	404	RCT	Depression	Usual care	Behavioral activation	12–24; not specified	Trained care coordinators	Individual; face to face	59	Type 2 diabetes
Kaur et al. (2018) India 2018	In patients	60	RCT	Depression anxiety	Usual care	Counseling	2; not specified	Trained researchers	Individual; face to face	100	Cervical cancer
Sasikumar (2017) India 2017	Community	38	RCT	Depression anxiety | mindfulness	Usual care	Mindfulness	1; not specified	Trained researchers	Not specified; face to face	60	Type 2 diabetes
Valsaraj et al. (2016) India, 2016	Out patients	67	RCT	Depression anxiety	Non-directive counseling	Cognitive behavioral therapy	10; 50–60 min	Not specified	Individual; face to face	30	Chronic kidney disease
Pathak et al. (2013) India, 2013	In Patients	100	Quasi-experimental	Depression anxiety	Usual care	Counseling	4; 30–40 min	Not specified	Individual; face to face	54	Cancer

### Inclusion criteria

#### Effectiveness

We included studies focusing on the treatment of depression (where the diagnosis of depression was made using a validated assessment instrument or diagnosed using any standardized diagnostic criteria) in people with NCDs (i.e. cardiovascular disease, type 2 diabetes, chronic obstructive pulmonary disorder, stroke, and cancer), adults (age ≥18 years), living in South Asia (i.e. Afghanistan, Bangladesh, Bhutan, India, Maldives, Nepal, Pakistan, and Sri Lanka) (South Asia, 2020). We included studies where the intervention was psychotherapy (e.g. counseling, cognitive behavior therapies, or behavioral activation). We included randomized controlled trials and quasi experimental studies The primary outcome was defined as the severity of depressive symptoms defined by scores on any standard depression scale; and we considered the prevalence of the depressive disorder, as defined by scores above a cut point for abnormality. Health-related quality of life, anxiety, adherence to treatment, cost-effectiveness, and adverse events were also included as secondary outcomes.

#### Barriers and facilitators to intervention development

For the barriers and facilitators we used the same inclusion criteria as for the effectiveness studies. However, we also included qualitative, quantitative studies and mixed-methods studies applied to stakeholders (therapist, healthcare providers, family members, and policymakers) as well. We summarized the number of barriers and facilitators for the implementation of psychological interventions. We used the theoretical domain framework (TDF) to code the extracted barriers and facilitators into domains (Atkins et al., 2017). The TDF contains 14 domains, which offer a theoretical perspective on the cognitive, affective, social, and environmental influences on behavior (Cane et al., 2012).

#### Policy level barriers and facilitators

Policy documents (legislation, strategy, and policy) related to mental health and/or NCDs in Bangladesh and Pakistan; articles (commentaries, research, etc.) about current legislation identified by the search within the last 5 years.

### Screening and study selection

Citations and available abstracts of the search results were uploaded into EndNote (version X9) and screened for potential eligibility in two stages. In the first stage, two independent reviewers screened titles and abstracts using Covidence (Melbourne, Australia). Discrepancies were resolved through consensus.

In the second stage, the full texts of potentially eligible studies were retrieved and assessed for eligibility by two independent experienced reviewers. For studies excluded at this stage, a reason for exclusion was recorded. Discrepancies were resolved through consensus. For included studies, multiple reports from the same study were linked.

### Data extraction

#### Effectiveness

Data for the assessment of study quality and evidence synthesis were extracted into an Excel database using a tailored and pre-piloted data collection template based on the Cochrane Consumers and Communication Group’s Data Extraction Template for Cochrane Reviews (Ryan et al., 2016). Data were extracted by one researcher and then checked independently by a senior researcher. Discrepancies were resolved by consulting a third reviewer. Missing data were requested from the study authors. We extracted data on the study population, country, setting, study design, intervention aim, intervention groups, primary outcome measure (reduction of depressive symptoms), secondary outcome measures (Health-related quality of life, anxiety symptoms, adherence to treatment, cost-effectiveness and adverse events), type of psychological intervention, comparator and duration of the intervention.

#### Barriers and facilitators for intervention development

Data on the individual and organizational level barriers and facilitators for intervention implementation were extracted using the TDF which offers an overarching theoretical framework that integrates constructs from multiple theories (Cane et al., 2012).

#### Policy level barriers and facilitators

We extracted data on (1) Summary of the policy/strategy/document; (2) Mentions and details of mental health In documents related to NCDs; (3) Evidence of integration of NCD and mental health care; (4) References and details on psychological interventions; (5) Service delivery/implementation details of psychological interventions; (6) Barriers to integration of psychological interventions in NCD care; (7) Facilitators of integrating psychological interventions in NCD care.

### Risk of bias (quality) assessment

#### Effectiveness

To assess the quality and risk of bias of randomized controlled trials we used the Cochrane Risk of Bias tool (RoB) 2.0 for the main outcome (depression) (Higgins et al., 2011), which assesses the following domains: (1) Sequence generation (selection bias); (2) Allocation concealment (selection bias); (3) Blinding of participants and personnel (performance bias); (4) Blinding of outcome assessment (detection bias); (5) Incomplete outcome data (attrition bias); (6) Selective outcome reporting (reporting bias); (7) Other potential sources of bias. We derived an overall summary RoB judgment (low; some concerns; high) for each specific outcome, whereby the overall RoB for each study was determined by the highest RoB level in any of the domains that were assessed. Any discrepancies in judgments or justifications for judgments were resolved by discussion to reach a consensus between two review authors, with a third review author acting as an arbiter if necessary (Higgins et al., 2011).

#### Barriers and facilitators

We assessed the RoB in the papers assessing barriers and facilitators using the Mixed Methods Appraisal Tool (MMAT) (Hawker et al., 2002; Hong et al., 2018). The included RCTs were evaluated again using the MMAT to reflect the quality of the study to evaluate barriers and facilitators for the implementation, rather than the effectiveness. We did not conduct a quality assessment or RoB for the identified policy level documents.

### Data synthesis

We conducted a narrative synthesis of the findings from the included studies (Campbell et al., 2020). For the effectiveness of interventions, we included the number of studies and the type of psychological interventions, the duration and effectiveness of the intervention, and the outcome measurements. We conducted a meta-analysis assessing the differences in mean values on depression for each arm of the RCTs. Standardized mean differences (mean/S.D.) between arms were calculated as the primary estimates of effect. We conducted analysis on short term outcomes <6 months and long term outcomes >12 months. If multiple measures were available before 6 months, the latest assessment before 6 months was selected. If they were multiple assessments after 12 months we used the last available measure.

For the barriers and facilitators, we synthesized the information using the TDF to code the extracted barriers and facilitators into 14 domains (Michie et al., 2005). For policy, we conducted a narrative synthesis of the included documents in each country.

## Results

**Figure 1.**
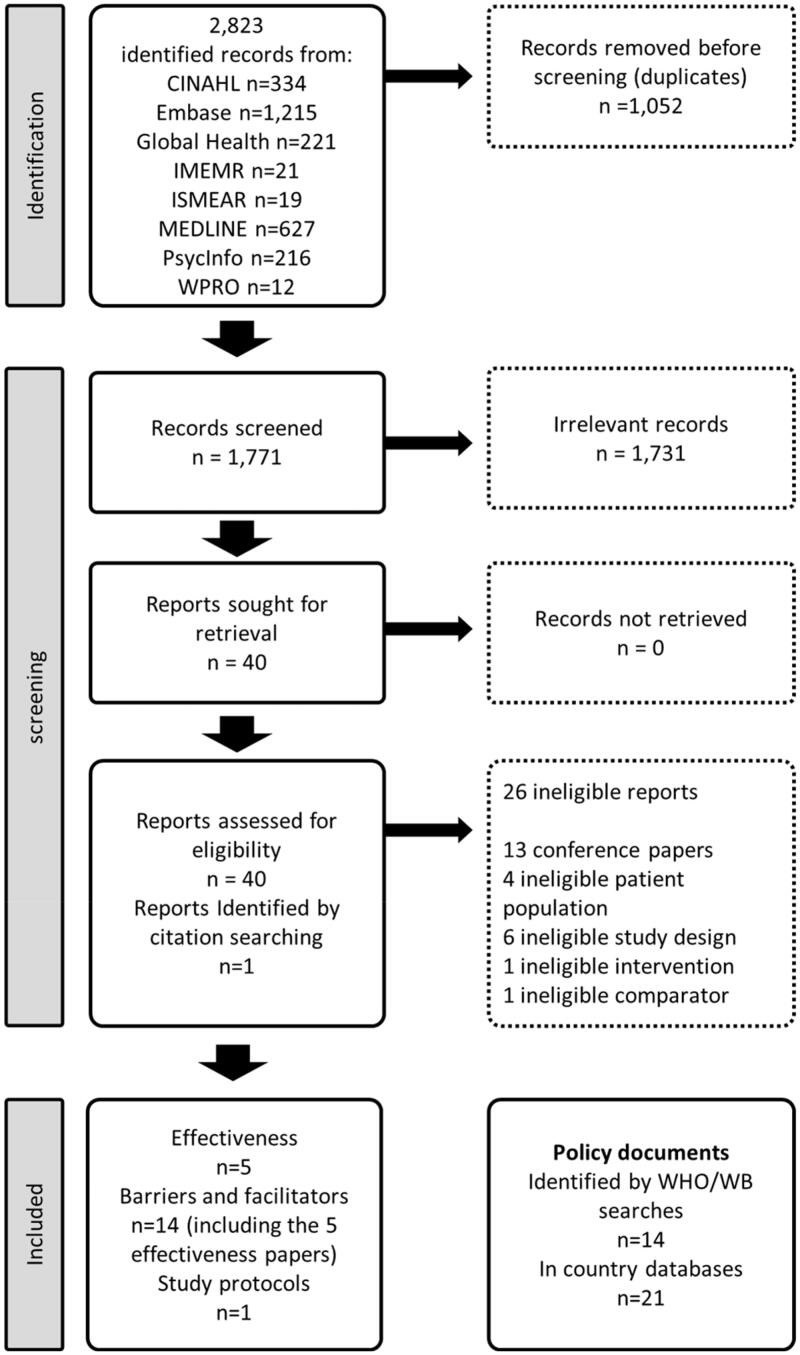
PRISMA flow diagram.

As seen in Figure 1, we identified 2823 records, which were reduced to 1771 after duplicates were removed and hand searching identified 1 record. We found 34 documents related to policy. All of the studies that were eligible for effectiveness (*n* = 5) were included in the barriers for implementation analysis (*n* = 14).

From the 1771 papers that were initially identified, 40 met our inclusion criteria for full text screening, and we included one additional study found as a reference. From the reports assessed for eligibility, 27 were ineligible, the reasons for exclusion are reported in Figure 1. In addition, we found a relevant protocol published in 2020, which was not included in the analysis (Chandra et al., 2020).

### Effectiveness

Five experimental studies, evaluating the effectiveness of psychological interventions in people with NCDs and depression were included (Table 1). Four were randomized controlled trials and one was a quasi-experimental study (pre-test post-test control group design). All the studies were conducted in India. One study was conducted in the community (Sasikumar, 2017) and four in hospital settings [two in inpatients (Kaur et al., 2018; Pathak et al., 2013) and two in outpatients (Ali et al., 2020; Valsaraj et al., 2016)]. One study was exclusively on women and the other four included between 30 and 60% of women.

All five studies used different outcome measures for depressive symptoms, which had been validated for the population where the studies were conducted. None of the studies reported depression remission post-trial. Ali et al. (2020) used a reduction in Symptom Checklist Depression Scale (SCL-20) and Patient Health Questionnaire-9 score (PHQ-9). Sasikumar (2017) used the Center for Epidemiologic Studies Depression Scale (CES-D), Kaur et al. (2018) used the Beck Depression Inventory, Valsaraj et al. (2016) used the Hospital Anxiety and Depression Scale (HADS), and Pathak et al. (2013) used the Beck Depression Inventory (BDI-II). Four studies reported anxiety as a secondary outcome (Pathak et al., 2013; Sasikumar, 2017; Valsaraj et al., 2016).

### Study characteristics

Two studies were conducted on people with type-2 diabetes (Ali et al., 2020; Sasikumar, 2017), two on people with cancer (Kaur et al., 2018; Pathak et al., 2013), and one on people with chronic kidney disease (Valsaraj et al., 2016). The intervention was delivered by trained researchers in two studies (Kaur et al., 2018; Sasikumar, 2017), non-physician care coordinators in one study (Ali et al., 2020) while three studies did not report who delivered the interventions (Pathak et al., 2013; Valsaraj et al., 2016). As seen in Table 1, two studies included counseling as psychotherapy (Kaur et al., 2018; Pathak et al., 2013), one CBT (Valsaraj et al., 2016), one Mindfulness (Sasikumar, 2017), and one behavioral activation (Ali et al., 2020). The number of sessions for various psychotherapies ranged from 1 (Sasikumar, 2017) to 24 (Ali et al., 2020). Four studies reported short term outcomes (6 months or less) and one reported long term outcomes (more than 12 months) (Ali et al., 2020).

#### Depressive symptoms (primary outcome)

**Figure 2.**
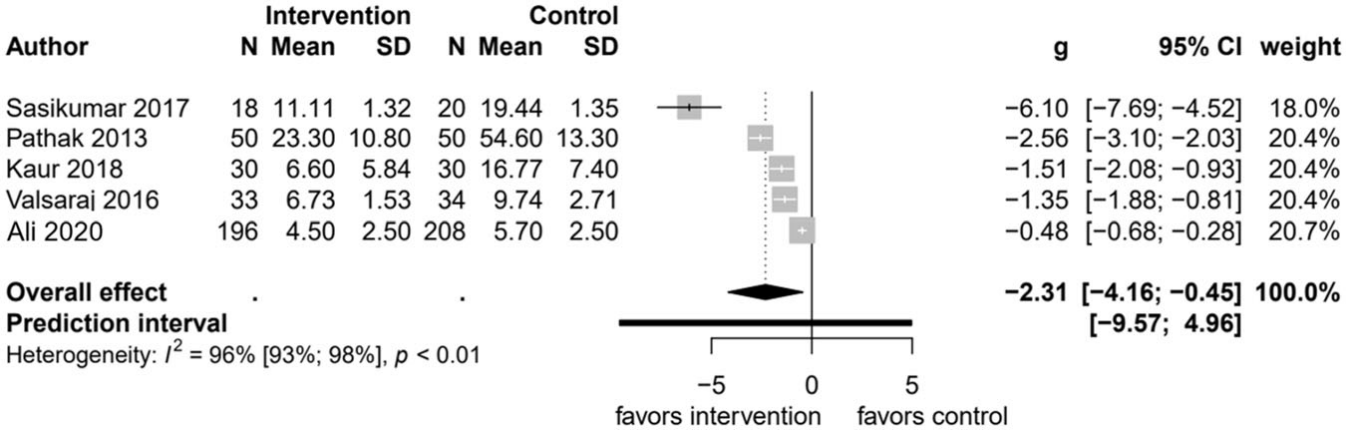
Forest plot comparing changes in depressive symptoms between psychological interventions and treatment as usual at endpoint.

None of the studies reported depression as a binary outcome. All interventions were demonstrated to be effective in reducing depressive symptoms in the short (6 months or less) and long term (more than 12 months) (Figure 2), with larger effect sizes in the four studies with smaller populations.

As seen in Figure 2, five studies reported the effectiveness of psychotherapy for depression as compared with usual care. There were a total of 669 participants in these studies, the majority (404) of participants were from the Ali et al study (Ali et al., 2020). The pooled standardized mean difference comparing psychological interventions with usual care was −2.31 (95% CI, −4.16 to −0.45; *p* = .015). Heterogeneity was observed among studies, with Cochran’s *Q* of 103.1 (*p* < 0.001), *I*^2^ of 96.0%, and *τ*^2^ of 4.3. Due to the high heterogeneity and differences between the type of psychotherapy and NCD, the results of the forest plot should be used descriptively.

#### Anxiety symptoms (secondary outcome)

**Figure 3.**
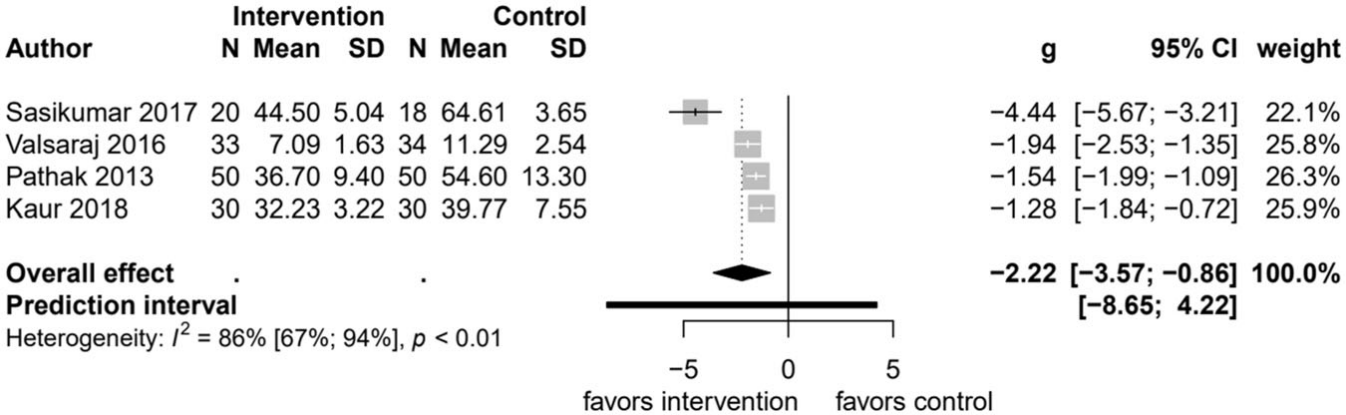
Forest plot comparing changes in short term anxiety symptoms between psychological interventions and treatment as usual.

Only four studies reported anxiety symptoms as an outcome (Figure 3), all the studies showed the psychological intervention to be effective to reduce anxiety symptoms. There were a total of 265 participants in these four studies. The intervention by Sasikumar (2017) showed the largest standardized effect size of −4.44 (95% CI, −6.67 to −3.21), and the pooled standardized mean difference was −2.22 (95% CI, −3.57 to −0.86; *p* = .001), with Cochran’s *Q* of 22.1 (*p* < 0.001), *I*^2^ of 86.5%, and *τ*^2^ of 1.7. Due to the high heterogeneity and differences between the studies, the results of the forest plot should be used in a descriptive way.

#### Adherence to treatment, cost-effectiveness, adverse events, and health related quality of life

None of the studies reported adverse events as an outcome and only the study by Ali et al. (2020) measured quality of life as an outcome, however, it was not reported in the results.

### Risk of bias

**Figure 4.**
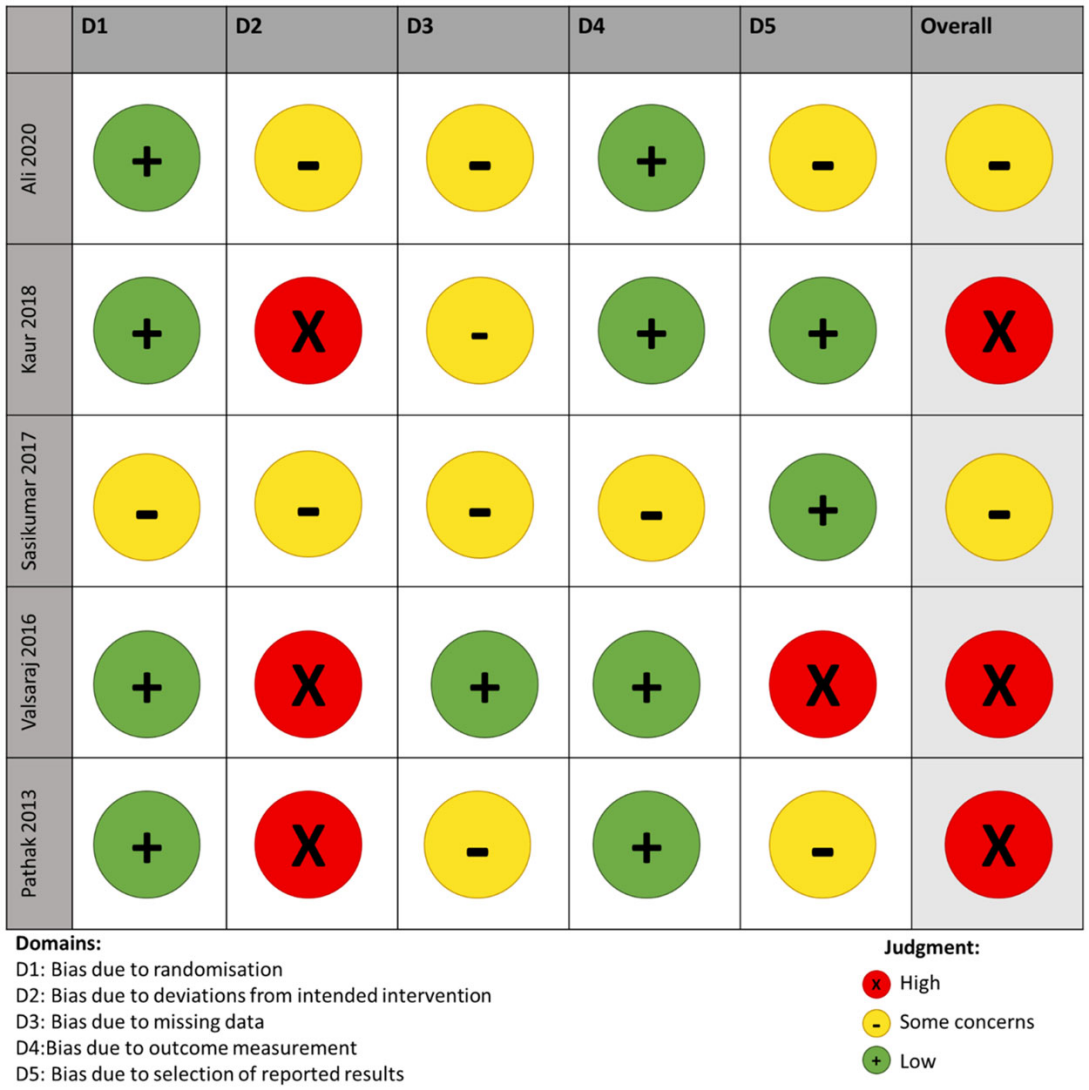
Cochrane risk of bias assessment presented as a domain across all included effectiveness studies.

Overall, the quality of the included studies was not optimal. As seen in Figure 4, only one of the five studies only met one of the criteria and none met all the criteria. In addition, all the studies had at least “some concerns” in the domain of “bias due to deviation of intended intervention.”

### Individual and organizational barriers and facilitators

We identified 14 studies, containing data that could support adaptation and implementation (Table 2). All studies were conducted in India except one multi-country study performed in Bangladesh and Pakistan. Seven studies reported qualitative methods, there were two quantitative non-randomized reports, four RCTs, and one case report. Quality was scored as mostly poor on the Hawker et al. (2002) scale (Hawker et al., 2002), and mostly adequate on the MMAT (Hong et al., 2018). Reporting of the details of the implementation was limited. We identified seven barriers to intervention delivery, of which five related to practitioner and patient barriers, and two related to intervention design. We found that the lack of availability of intervention materials in patients’ first language was a significant barrier to engagement. Pessimism around beliefs about capabilities in older patients as well as perceived capacity for engaging with the intervention because of anxiety or memory/attention limitations, staff familiarity with talking therapies, stigma around mental health, and a failure to consider depression as something distinct from NCD were barriers among patients and practitioners. Among practitioners, time constraints and a lack of knowledge around mental health were also identified as barriers.

**Table 2.** Studies included in the individual and organizational barriers and facilitators analysis.

Author	Design	Theoretical framework	Sample size	Overall quality assessment (Hong et al., 2018)
Cohen et al. (2018) India 2018	Qualitative	Not mentioned	20	High
Datta et al. (2016) India 2016	RCT	Not mentioned	107	Some concerns
Rao et al. (2016) India 2016	Qualitative	Grounded theory	82	High
Dias et al. (2017) India 2017	Qualitative	Not mentioned	NA	Low
Dias et al. (2019) India 2019	Qualitative	Not mentioned	32	High
Gomes et al. (2016) India 2016	Case study	IPT principles.	1	Some concerns
Johnson et al. (2020) India 2019	Qualitative	Not mentioned	62	High
Kaur et al. (2018) India 2018	Quasi-experimental	Not mentioned	60	Some concerns
Ali et al. (2020) India 2020	RCT	Not mentioned	404	High
Pathak et al. (2013) India 2013	Quasi-experimental	Not mentioned	100	Some concerns
Reynolds et al. (2017) India 2018	Qualitative	Not mentioned	NA	Low
Sasikumar (2017) India 2017	RCT	Not mentioned	38	High
Valsaraj et al. (2016) India 2016	RCT	Not mentioned	80	High
Wright et al. (2020) Bangladesh and Pakistan 2020	Qualitative	COM-B	67	High
Mixed methods appraisal tool (Hong et al., 2018)				

We identified five facilitators, of which three related to patient or practitioner and two related to intervention design. Intervention training focused on knowledge and skills was seen as effective to enhance the delivery among practitioners, and knowledge of local community assets that supported intervention tailoring for participants was identified as an important facilitator. Among patients, a supportive community environment, with family engagement and resources, was found to support intervention implementation. In terms of interventions, utilizing in clinical settings reduced interruptions, and simple visual materials increased engagement.

### Quality appraisal

Eight studies were categorized as “high” quality, four as “some concerns” and only two of them as “low” quality.

### Policy level barriers and facilitators

We found 35 documents (15 Bangladesh and 20 Pakistan) that included policy information about barriers and facilitators to implement psychological interventions. The full list of documents can be found in the Supplementary Appendix.

#### Policy environment in Bangladesh for integration of mental health into NCD care

Bangladesh has a pluralistic health system, with government health care provided at district level hospitals, upazila (sub-district) level health complexes, union health and family welfare centers, and ward level community clinics. Its policies and laws, however, are centralized.

The Bangladesh WHO Special Initiative for Mental Health Situational Assessment (2020) is positive overall about the policy environment of mental health (WHO, 2020). New legislation specifically on mental health includes the Bangladesh Mental Health Act 2018 (replacing the 1912 Lunacy Act) which encourages provisions for health and rehabilitative services for those with a mental disorder, a new Mental Health Policy, and a Mental Health National Strategic Plan 2020–2030 (yet to be released). The documents are being developed to “establish a comprehensive, multi-sectoral, integrated and responsive system to ensure universal access to and effective utilization of quality mental health and psychosocial services and information system” (WHO, 2020). Other policy documents related to healthcare generally highlight the importance of mental health and its integration into universal health coverage (UHC). Both the multisectoral action plan for the prevention and control of NCDs 2018–2025 (Dhaka: Non-communicable Disease Control Programme, Directorate General of Health Services, 2018) and the Health Nutrition and Population Strategic Investment Plan (HNPSP) 2018–2021 (Ministry of Health and Family Welfare Government of the People Republic of Bangladesh, 2016) refer to SDG 3.4 “to promote mental health and wellbeing” and both plans outline working toward achieving UHC and improving health care at primary care level.

While policies increasingly recognize the importance of integrating mental health into the health systems the actual implementation of mental health services and psychological support appears to be lacking. This is iterated by the mid-term review of the Health, Population and Nutrition Sector Program 2017–2022 (HNSIP) plan conducted in 2020, which found that while a good start to introducing NCDs had been made, with a few notable exceptions mental health had been largely neglected (Ministry of Health and Family Welfare Government of the People Republic of Bangladesh, 2016). However, there is also a lack of funding, training in mental health, and resources. The WHO Special Initiative for mental health Situational Assessment notes on the ground there is a lack of psychosocial interventions outside the national psychiatric hospital and the medical teaching hospital (WHO, 2020). There is a lack of psychologists and qualified staff to provide psychosocial interventions as well as stigma meaning people do not seek care.

Mental health is clearly on the agenda in Bangladesh, and there is a shift to a multi-sectoral approach with the aim to incorporate mental health provision into universal health coverage. However, there are few specific mentions of psychological interventions specifically. Furthermore, there remains a lack of health care staff trained in mental health and providing psychosocial support, services are severely underfunded and there are high levels of inequity and stigma regarding mental health.

#### Policy environment in Pakistan for integration of mental health into NCD care

Pakistan operates as a three level public healthcare system; with health systems and related policies in Pakistan being largely decentralized meaning significant differences across the 4 provinces (Punjab, Sindh, Khyber Pakhtunkhwa, Baluchistan) and one federal territory (Islamabad Capital territory). The existing National policies do include MH. For example, the National 5-year health plan aligns with the SDGs and plans for UHC with mental health being included (Ministry of Planning, Development & Reform, 2018). In the National Health Vision 2016–2025 mental health is mentioned in the essential health service package (EHSP) and as a focus in cross-sectoral actions to advance health. However, for both documents, there are few details about implementation. The recent National Action Framework for Non-Communicable Disease and Mental Health 2021–2030 (Inter-Ministerial Health & Population Council, 2022), however, identifies core strategic areas for interventions related to the prevention and control of NCDs and mental health with tangible targets for morbidity, mortality and risk factors. While the integration of MH into NCD care is not explicitly mentioned it does advocate the integration of MH (and psychological support specifically) into PHC, as part of UHC and ESP. Furthermore, strategic actions include revisiting policy and establishing a multi-sectoral high-level committee to steer reforms related to NCD and mental health.

At a provincial level MH is at least mentioned in all 5 province/territories’ policies examined, though none explicitly discuss psychological interventions. Documents relating to Punjab and Sindh have some details of implementation plans. The Sindh Health Sector Strategy includes strategic actions to cover mental health under EHSP and as part of NCD care. They include: formulating the EHSP in secondary care, boosting health worker skills, developing links with mental health service packages increase budgetary support. The Punjab Provincial Strategic Plan for NCDs and MH has a section on MH care and an NCD and Mental Health Activity Plan with specific targets in terms of implementing MH care at a district level and plans for training of healthcare workers. Pharmacological treatments, “intervention guidelines” and referrals to specialists are all mentioned as aspects of outpatient and community care. It also highlights the current lack of guidelines, training, and specialists in mental health. While Khyber Pakhtunkhwa’s (KP) health policy and health sector strategic plans include mental health in EHSP there are no details about implementation. KP does have a Mental Health Act 2017 that discusses access to treatment and care, although psychological interventions are not mentioned explicitly. The Baluchistan Health Policy has an emphasis on UHC and inter-sectoral approach, but mental health is not mentioned. The Health Sector Strategy does include promoting mental health and well-being as an objective with scaling up of services as part NCD, the integration into PHC, and surveillance all mentioned. However, there is a lack of details as to what this means for mental health. The strategy also highlights several weaknesses in the health system and with resources.

Overall, in Pakistan, the recognition for an incorporation of mental health services as part of EHSP, often with NCDs, is encouraging. The level of detail in planning and implementation vary across regions. Additionally, there are weaknesses in health systems, a lack of funding for services, and a lack of training and specialists.

#### Policy level barriers and facilitators for implementation

In both Bangladesh and Pakistan, the policy environment is generally conducive to incorporating mental health services into healthcare and there is an important recognition that they should be part of essential services. However, there are major challenges in terms of training and resources. Furthermore, there are few specific implementation plans or mention of specific disorders (such as depression) and specifically psychological interventions. Table 3 highlights the specific facilitators and barriers to implementing psychological interventions to treat people with NCDs.

**Table 3.** Policy barriers and facilitators for implementing psychological interventions to treat depression in people with NCDs.

	Pakistan	Bangladesh
Facilitators		
	Mental health is mentioned in policies, generally a positive policy environment	Generally a positive policy environment of MH with the new strategy and plan being approved which aims to facilitative cross-sectoral action and aiming to ensure universal access to psychosocial mental health care
	There is a move and planned actions toward UHC and Mental Health is included in the ESP	There is a move and planned actions toward UHC and Mental Health is included in the ESP
	Pakistan aligning itself with SDGs, including SDG 3.4	The policies and plans for NCD and MH very much align with the SDGs including SDG 3.4
	Pakistan has worked with the WHO on the MHGAP	Bangladesh has worked with the WHO to implement the MHGap
	Increased focus on inter-sectoral approach and “health in all” policies	There has been a long-standing commitment to integrate mental health into primary care
	Examples of policies with clear plans: Punjab for example have targets and plans for MH care	There has been a long-standing commitment to integrate mental health into primary care
		A general increased recognition of the importance of mental health
		There are advocacy groups and NGOs working in the area of mental health
Barriers		
	Differences across provinces: will depend on regional political will and budgets	Mental Health is not being implemented in the ESP in practice
		Very little mention of MH in NCD strategy and plan
	Lack of integration of MH and NCD care	No apparent integration of MH into NCD care
	MH is missing from several of the health policies or mentioned in very general terms	MH discussed in very general terms
	Little mention of psychological interventions	Negligible mention of psychological interventions as part of MH care (apart from 1/2 examples) and no concrete plans to train or roll them out
	Poor referrals between sectors and levels	Poor referrals between sectors and levels
	Lack of training and providers	Lack of training and providers
	Very little budget/lack of resources	Very little budget/lack of resources

## Discussion

We found a limited amount of interventions to address depression in people with NCDs in South Asia and the overall quality of the studies was poor. The few available interventions showed that psychological interventions are effective to improve depressive and anxiety symptoms in this population. However, due to the high heterogeneity and low quality of the available studies, it is not possible to determine if psychological interventions (pooled effect-size) is effective to treat depression in people with NCDs in South Asia. We found multiple barriers to deliver these interventions both at an individual, organization, and policy level.

### Effectiveness of interventions

Similarly to interventions developed in other regions of the world (Dickens et al., 2013; Smith et al., 2014), all the psychological interventions in this review showed to be effective to reduce depressive symptoms in this population. Given the small sample size and the high proportion of studies found to have “some concerns” or “high” risk of bias, there is a need to evaluate the effectiveness and cost-effectiveness of well-designed studies with adequate samples to provide substantive evidence for the use of psychological interventions in this population. It is important to highlight the high heterogeneity in between the studies, which may be related to differences in the outcome measurements, type of psychological interventions, and NCDs. The results of the meta-analysis should be used as a descriptive of the independent studies.

### Barriers and facilitators for implementation

In line with other interventions in South Asia, knowledge about the disease, community assets, and supportive family engagement were found as facilitators for the delivery of interventions (Joseph et al., 2019; Morrison et al., 2019), and may be effective strategies to enhance the effectiveness and acceptability of interventions in this setting (Bernal et al., 2009). Simplifying intervention materials were also found to be effective to improve engagement, and reach an illiterate population (Sharma et al., 2016).

The availability of resources in the multiple languages spoken in each region of South Asia has been consistently found as a barrier to the implementation of interventions in these settings (Ahmed et al., 2018). Translating the materials and having facilitators that speak multiple languages are strategies that have been used by different research groups to overcome this challenge and should be considered in the implementation of further interventions (Ahmed et al., 2018).

At a policy level, there is clear political will to have mental health on the agenda and an increasing recognition that mental health services are essential in both countries. In Bangladesh there is a strong commitment to mental health reform, the development of the new mental health policy (though not yet released) is testament to this. In Pakistan, there has been an increased focus on inter-sectoral health care and the importance of mental health. Both countries include at least some level of MH care on their ESP. This is indeed encouraging, however, the available documents tend to lack details as to specific plans for the integration of MH care, and specifically into NCD care. Furthermore, there is little mention of psychological interventions. Other gaps identified around implementation include heavily bureaucratic systems, lack of funding, lack of training and staffing levels in both countries. The WHO Europe have recommended some pathways to the integration of mental health within the NCD agenda which both countries could build; however do require strong leadership, finances, and monitoring and evaluation of needs and impact (Integrating the prevention, treatment and care of mental health conditions and other NCDs within health systems, 2019). The WHO also recognizes psychological interventions as effective and potentially scalable (WHO, 2017). On a practical level, any integration of psychological interventions into NCD care would need logistical support. However, it could be a cost-effective option that would complement current mental health policy. Both the Bangladesh and Pakistan governments have worked with the WHO to implement WHO MHGap programme and thus have demonstrated an ability to work with international agencies on mental health implementation. As the growing threat of NCDs (including mental health) is increasingly recognized it is clear that funding priorities and allocations need to better align to address these issues (Jailobaeva et al., 2021).

### Limitations

The few studies that evaluated the implementation of interventions for depression and NCDs in these populations did not follow a standardized framework to evaluate the implementation. Because of time and resources constraints, we used a limited search including only eight databases for effectiveness and barriers and facilitators studies, we did not include local or large multidisciplinary databases (e.g. PakMediNet, Scopus, Web of Science Core Collection), For the policy search, we may have missed some documents as it is possible not all relevant documents were available on the selected websites or were not yet available to the general public via the web. In addition, we didn’t conduct a comprehensive search of grey literature (e.g. theses, conferences, trials registries). Another limitation is that we only evaluated studies in South Asia, studies from other LMICs with similar contexts could have also provided insight into barriers to the implementation of these interventions.

## Conclusion

We found a limited amount of studies testing the effectiveness of psychological interventions for depression in people with NCDs, all of the independent studies reported to be effective to reduce depressive symptoms in people with different NCDs. However, due to the small sample size and high risk of bias of the studies more high quality trials are needed to evaluate the effectiveness and implementation outcomes of psychological interventions in this context. We identified context-specific barriers and facilitators that should be considered while developing and implementing interventions in this context.

## Author’s contributions

GZ, DE, IK, JW, HJ, and NS designed the study. JW designed and conducted the literature searches. All authors except JW were involved in the screening. GZ, IK, CK, and FA did data extraction. GZ, HJ, and IK drafted the article. All the co-authors critically revised the article. All authors approved the final version of the manuscript.

## Protocol publication

The protocol has been published at the International prospective register of systematic reviews (PROSPERO) and is accessible at https://www.crd.york.ac.uk/prospero/display_record.php?ID=CRD42020220287 (Zavala et al., 2020).

## Supplementary Material

Supplemental Material

## Data Availability

The systematic searches are available as a Supplementary Appendix.
