# Supplementary material for: Effectiveness and implementation of psychological interventions for depression in people with non-communicable diseases in South Asia: Systematic review and meta-analysis
Source: Int J Ment Health. 2023 Apr 24;52(3):260–84. doi: 10.1080/00207411.2023.2202431 (PMC10461698; doi:10.1080/00207411.2023.2202431)
Supplement: Supplemental Material [file MIMH_A_2202431_SM3375.zip › Appendix 1.docx]

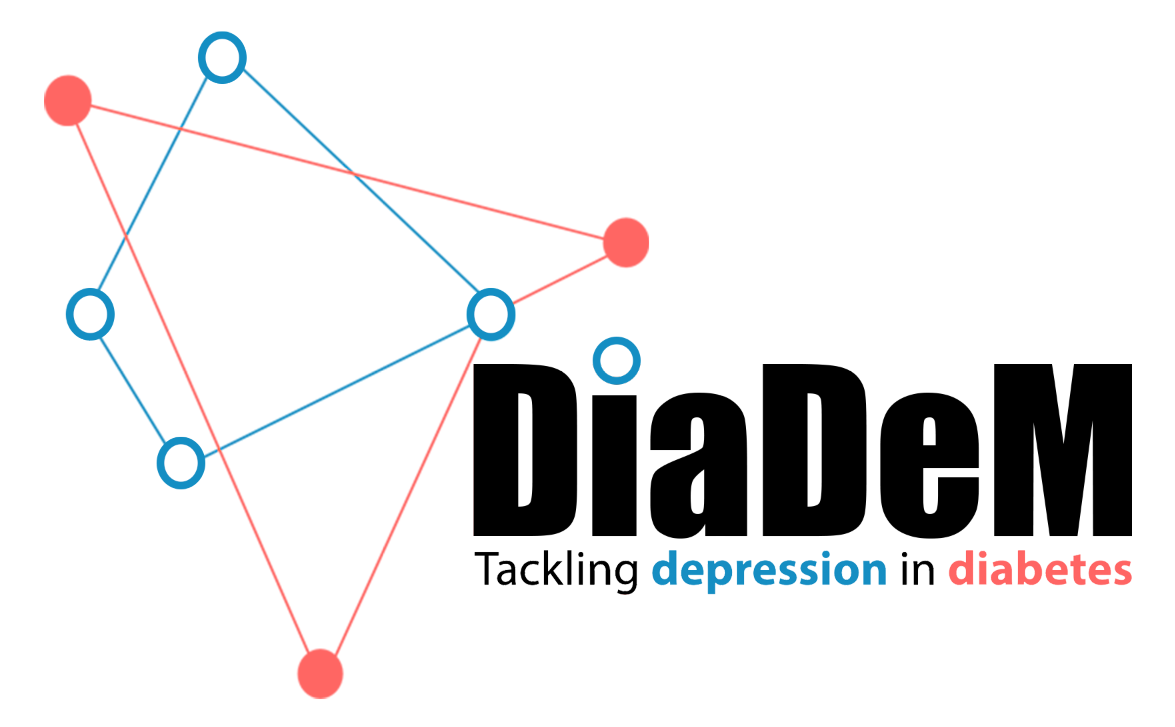


#

#

**Psychological interventions for depression in people with non-communicable diseases in South Asia**

**Appendix 2. Search Strategies**

**Databases Searched (19-10-2020 and 05-05-2022):**

- Applied Social Sciences Index and Abstracts (ASSIA)‎ (ProQuest) 1987- present
- CINAHL (EBSCOhost) 1981- present
- Embase Classic+Embase (Ovid) <1947 to 2020 October 15>
- Global Health <1910 to 2020 Week 41>
- IMEMR Index Medicus for the Eastern Mediterranean (via WHO Global Health Index Medicus), all available years.
- ISMEAR Index Medicus for the South East Asia Region (via WHO Global Health Index Medicus), all available years.
- Ovid MEDLINE(R) and Epub Ahead of Print, In-Process & Other Non-Indexed Citations and Daily <1946 to October 15, 2020>
- APA PsycInfo (Ovid) <1806 to October Week 2 2020>

# Search Strategies

Each database was searched using two search strategy, a main search of S Asian literature supplemented by a second search focussed on Bangladesh/Pakistan studies

• Search 1 Concepts: Behavioural Activation/Psychological interventions AND NCDs AND Depression AND S Asia

• Search 2 Concepts: Behavioural Activation /Psychological interventions AND NCDs AND Mental Disorders AND Bangladesh/Pakistan

Applied Social Sciences Index and Abstracts (ASSIA)‎ (ProQuest) 1987+ Search 1

Date searched: 05/05/2022

Records found: 95

S17 16 and 13 and 12 and 8 95

S16 15 or 14 22,774

S15 noft(Bhutan or afghanistan or maldives or nepal or "sri lanka" OR Bhutani or afghanistani or nepalese or "sri lankan" or bangladesh or pakistan or bangladeshi or pakistani or "south asia" or "south asian") 11,032

S14 ti(Indian or Indians or India ) OR ab(Indian or Indians or India ) OR loc(Indian or Indians or India ) 13,064

S13 noft(depressive or depression or depressed or dysthymia or mood or moods or "mental health" or "emotional trauma " or "psychological trauma" or "common mental disorder*") 168,696

S12 9 or 10 or 11 118,891

S11 noft((brain or cerebral or cerebellum or intracranial or intracerebral or subarachnoid) and (ischemia or ischaemia or ischemic or ischaemic or infarction or thrombosis or embolism or occlusion or hemorrhage or haemorrhage or hemorrhages or haemorrhages or hematoma or haematoma or hematomas or haematomas or bleed or bleeds or bleeding)) OR noft("transient ischemic attack " or "transient ischaemic attack " or noncommunicable or non-communicable or non-infectious or non infectious) OR noft(cancer or cancers or cancerous or carcinoma or tumor or tumors or tumour or tumours or neoplasm or neoplasms or malignant or malignancy or malignancies) 45,674

S10 noft((airway or airways or pulmonary or airflow or airflows or lung or lungs) and (obstruct or obstructed or obstruction or obstructing or emphysema)) OR noft((bronchitis or respiratory or respiration or lung or lungs) and (condition or conditions or disease or diseases or symptom or symptoms or problem or problem or long-term or chronic or chronically) ) OR noft(stroke or poststroke or post-stroke or cerebrovascular or "brain vasculitis" or "cerebral vasculitis" or apoplexy) 17,644

S9 noft(("long term" or chronic) near/3 (disease or diseases or illness or illnesses or condition or conditions or disorder or disorders)) OR noft(diabetes or diabetic or Aneurysm or aneurysms or Atherosclerosis or heart or cardiac or valvular or coronary or arterial or vascular or hypertension) OR noft(Cardiovascular or cardiomyopathy or endocardium o r endocarditis or pericardium or pericarditis) 69,325

S8 1 or 2 or 3 or 4 or 5 or 6 or 7 348,280

S7 noft(anxiety and (manage OR managed OR management OR managing)) OR noft(multimodal or multi-modal or "combined modal" or "combined modality") OR ti(health and (education or educational or educate or promote or promotion or promotional)) OR ab(health and (education or educational or educate or promote or promotion or promotional)) 66,286

S6 noft("third wave" or "cognitive restructure" or "cognitive restricturing " or "positive psychology" or reframe or reframed or reframing or re-frame or re-framed or re-framing) OR noft(reappraise or reappraisal or reappraised or "acceptance and commitment") OR ti(stress AND (inoculation OR manage OR managed OR management OR managing OR reduce OR reduced OR reducing OR reduction OR resist OR resists OR resisted OR resisting)) OR ab(stress AND (inoculation OR manage OR managed OR management OR managing OR reduce OR reduced OR reducing OR reduction OR resist OR resists OR resisted OR resisting)) 16,040

S5 noft("brief therapy" or "brief therapies" or relaxation or mindful or mindfullness or counseling or counselling or coaching) OR ti((cognitive or cognition or CBT or psychological) AND (intervention OR interventions OR program OR programs OR programme OR programmes OR therapy OR therapies)) OR ab((cognitive or cognition or CBT or psychological) AND (intervention OR interventions OR program OR programs OR programme OR programmes OR therapy OR therapies)) 88,587

S4 noft(psychotherapy or psychotherapeutic or psycho-therapy or psycho-therapeutic) OR ti((behavior or behaviour or behavioral or behavioural) and (intervention or interventions or program or programs or programme or programmes or therapy or therapies)) OR ab((behavior or behaviour or behavioral or behavioural) and (intervention or interventions or program or programs or programme or programmes or therapy or therapies)) 81,404

S3 noft("functional analysis") OR noft((gain or gains or gained or gaining or reappraise or reappraisal or reappraised) and (focus or focusing)) OR noft((psychoeducation or psychoeducational or psycho-education or psycho-educational) and (self or behavior or behaviour)) 5,503

S2 noft((activity or activities) and (schedule or scheduled or pleasurable or pleased or enjoy or enjoyed or enjoyment or reward or rewarding or rewarded or rewards)) OR noft((operant or instrumental) and (conditioning or learning)) OR noft(positive or coping or contingency or contingencies) 124,253

S1 noft((behavior or behavioral) and (activation or evaluation or evaluate or monitor or monitoring or train or training or treatment or treatments or contracting or modification or modify or modifying)) OR noft((behaviour or behavioural) and (activation or evaluation or evaluate or monitor or monitoring or train or training or treatment or treatments or contracting or modification or modify or modifying)) OR noft(reinforce or reinforcer or reinforcement or reinforcements or re-inforcement or re-inforcements) 96,894

Applied Social Sciences Index and Abstracts (ASSIA)‎ (ProQuest) 1987+ Search 2

Date searched: 05/05/2022

Records found: 63

S18 17 and 16 and 12 and 8 63

S17 noft(bangladesh or pakistan or bangladeshi or pakistani) 6,549

S16 13 or 14 or 15 419,004

S15 noft((PTSD or "psychological trauma" or psychotrauma or "combat disorder" or "war disorder" or psychotraumas or "combat disorders" or "war disorders") ) OR noft((bipolar or behavioral or behavioural or obsessive or compulsive or panic or mood or delusional) and (disorder or illness or disease or disorders or illnesses or diseases)) OR noft((trichotillomania or OCD or "obsessive-compulsive" or GAD or "stress reaction" or "acute stress" or neurosis or neuroses or neurotic)) 58,947

S14 noft((stress or cognitive or cognition or personality or "impulse control" or mood or paranoid or psychotic or neurologic* or nervous or nervous system or eating) and (disorder or disorders or disease or diseases or illness or illnesses or symptom or symptoms)) OR noft((post-trauma or posttrauma or post-traumatic or posttraumatic) and (stress or disorder or disorders)) OR noft("stress syndrome" or "distress syndrome" or "pain disorder" or "pain disorders" or dementia or alzheimer or alzheimers) OR noft(("substance abuse" or "substance use" or "drug abuse" or "drug use" or personality or sleep) and (disorder or disorders or syndrome or syndromes) ) 119,118

S13 noft(mental or mentally or psychiatric or psychological or psychologically or depression or depressive or depressed or MDD or anxiety or anxious or phobia or phobic or agoraphobia or dysthymia or ADNOS) OR noft(schizophrenia or schizophrenic or akathisia or acathisia or neuroleptic-induced or dissociative or dissociation) OR noft(somatization or somatisation or somatoform or somatic or hysteria or hysterical or briquet or multisomat* or MUPs or "medically unexplained") 371,328

S12 9 or 10 or 11 118,891

S11 noft((brain or cerebral or cerebellum or intracranial or intracerebral or subarachnoid) and (ischemia or ischaemia or ischemic or ischaemic or infarction or thrombosis or embolism or occlusion or hemorrhage or haemorrhage or hemorrhages or haemorrhages or hematoma or haematoma or hematomas or haematomas or bleed or bleeds or bleeding)) OR noft("transient ischemic attack " or "transient ischaemic attack " or noncommunicable or non-communicable or non-infectious or non infectious) OR noft(cancer or cancers or cancerous or carcinoma or tumor or tumors or tumour or tumours or neoplasm or neoplasms or malignant or malignancy or malignancies) 45,674

S10 noft((airway or airways or pulmonary or airflow or airflows or lung or lungs) and (obstruct or obstructed or obstruction or obstructing or emphysema)) OR noft((bronchitis or respiratory or respiration or lung or lungs) and (condition or conditions or disease or diseases or symptom or symptoms or problem or problem or long-term or chronic or chronically) ) OR noft(stroke or poststroke or post-stroke or cerebrovascular or "brain vasculitis" or "cerebral vasculitis" or apoplexy) 17,644

S9 noft(("long term" or chronic) near/3 (disease or diseases or illness or illnesses or condition or conditions or disorder or disorders)) OR noft(diabetes or diabetic or Aneurysm or aneurysms or Atherosclerosis or heart or cardiac or valvular or coronary or arterial or vascular or hypertension) OR noft(Cardiovascular or cardiomyopathy or endocardium o r endocarditis or pericardium or pericarditis) 69,325

S8 1 or 2 or 3 or 4 or 5 or 6 or 7 348,280

S7 noft(anxiety and (manage OR managed OR management OR managing)) OR noft(multimodal or multi-modal or "combined modal" or "combined modality") OR ti(health and (education or educational or educate or promote or promotion or promotional)) OR ab(health and (education or educational or educate or promote or promotion or promotional)) 66,286

S6 noft("third wave" or "cognitive restructure" or "cognitive restricturing " or "positive psychology" or reframe or reframed or reframing or re-frame or re-framed or re-framing) OR noft(reappraise or reappraisal or reappraised or "acceptance and commitment") OR ti(stress AND (inoculation OR manage OR managed OR management OR managing OR reduce OR reduced OR reducing OR reduction OR resist OR resists OR resisted OR resisting)) OR ab(stress AND (inoculation OR manage OR managed OR management OR managing OR reduce OR reduced OR reducing OR reduction OR resist OR resists OR resisted OR resisting)) 16,040

S5 noft("brief therapy" or "brief therapies" or relaxation or mindful or mindfullness or counseling or counselling or coaching) OR ti((cognitive or cognition or CBT or psychological) AND (intervention OR interventions OR program OR programs OR programme OR programmes OR therapy OR therapies)) OR ab((cognitive or cognition or CBT or psychological) AND (intervention OR interventions OR program OR programs OR programme OR programmes OR therapy OR therapies)) 88,587

S4 noft(psychotherapy or psychotherapeutic or psycho-therapy or psycho-therapeutic) OR ti((behavior or behaviour or behavioral or behavioural) and (intervention or interventions or program or programs or programme or programmes or therapy or therapies)) OR ab((behavior or behaviour or behavioral or behavioural) and (intervention or interventions or program or programs or programme or programmes or therapy or therapies)) 81,404

S3 noft("functional analysis") OR noft((gain or gains or gained or gaining or reappraise or reappraisal or reappraised) and (focus or focusing)) OR noft((psychoeducation or psychoeducational or psycho-education or psycho-educational) and (self or behavior or behaviour)) 5,503

S2 noft((activity or activities) and (schedule or scheduled or pleasurable or pleased or enjoy or enjoyed or enjoyment or reward or rewarding or rewarded or rewards)) OR noft((operant or instrumental) and (conditioning or learning)) OR noft(positive or coping or contingency or contingencies) 124,253

S1 noft((behavior or behavioral) and (activation or evaluation or evaluate or monitor or monitoring or train or training or treatment or treatments or contracting or modification or modify or modifying)) OR noft((behaviour or behavioural) and (activation or evaluation or evaluate or monitor or monitoring or train or training or treatment or treatments or contracting or modification or modify or modifying)) OR noft(reinforce or reinforcer or reinforcement or reinforcements or re-inforcement or re-inforcements) 96,894

CINAHL (EBSCOhost) 1981+ Search 1

Date searched: 05/05/2022

Records found: 240

# Query Results

S37 S17 AND S20 AND S31 AND S36 240

S36 S32 OR S33 OR S34 OR S35 172,091

S35 TI ( ("south asia*" or bangladesh* or pakistan*) ) OR AB ( ("south asia*" or bangladesh* or pakistan*) ) OR AF ( ("south asia*" or bangladesh* or pakistan*) ) 23,171

S34 TI ( (Bhutan* or afghanistan* or maldive* or nepal* or "sri lanka*" or india) ) OR AB ( (Bhutan* or afghanistan* or maldive* or nepal* or "sri lanka*" or india) ) OR AF ( (Bhutan* or afghanistan* or maldive* or nepal* or "sri lanka*" or india) ) 135,251

S33 TI ( (Indian or Indians) not ("west indian*" or "american indian*") ) OR AB ( (Indian or Indians) not ("west indian*" or "american indian*") ) OR AF ( (Indian or Indians) not ("west indian*" or "american indian*") ) 22,392

S32 (MH "Bangladesh") OR (MH "Bhutan") OR (MH "India") OR (MH "Nepal") OR (MH "Pakistan") OR (MH "Sri Lanka") OR (MH "Afghanistan") 59,289

S31 S21 OR S22 OR S23 OR S24 OR S25 OR S26 OR S27 OR S28 OR S29 OR S30 2,054,194

S30 TX (transi* n3 isch?em* n3 attack?) OR TX ( ((noncommunicable or non-communicable or non-infectious or non infectious) n3 (disease? or illness* or condition? or disorder?)) ) or TI (cancer* or carcinoma? or tumor? or tumour? or neoplasm? or malignan*) or AB (cancer* or carcinoma? or tumor? or tumour? or neoplasm? or malignan*) 646,255

S29 TX ( ((brain? or cerebr* or cerebell* or intracran* or intracerebral) n2 (isch?emi* or infarct* or thrombo* or emboli* or occlus*)) ) OR TX ( ((brain? or cerebr* or cerebell* or intracerebral or intracranial or subarachnoid) n2 (h?emorrhage? or h?ematoma* or bleed*)) ) 17,791

S28 TX ( ((long-term or Chronic*) n2 lung* n5 (condition* or disease* or symptom* or problem* or failure*)) ) OR TX ( (respiratory n2 (condition* or disease* or symptom* or problem*)) ) AND TX ( (stroke or poststroke or post-stroke or cerebrovasc* or brain vasc* or cerebral vasc* or apoplex*) ) 5,204

S27 TX ( ((long-term or chronic*) n2 (airway* or pulmonar* or airflow* or lung*) n2 Obstruct*) ) OR TX (pulmonar* n2 emphysem*) OR TX ( ((long-term or chronic*) n5 (bronchitis or respirat*)) ) 33,285

S26 TI (diabet*) or AB (diabet*) 228,014

S25 TI ( Aneurysm* or Atherosclerosis or Cardio* or endocard* or pericard* ) OR TI ( (heart or cardiac or valvular or coronary or arterial or vascular or hypertension) ) OR AB ( Aneurysm* or Atherosclerosis or Cardio* or endocard* or pericard* ) OR AB ( (heart or cardiac or valvular or coronary or arterial or vascular or hypertension) ) 670,221

S24 (MH "Neoplasms+") OR (MH "Diabetes Mellitus+") 796,252

S23 (MH "Stroke+") OR (MH "Pulmonary Disease, Chronic Obstructive+") OR (MH "Cardiovascular Diseases+") 664,699

S22 (MH "Diabetes Mellitus+") 182,209

S21 ( (MH "Chronic Disease") OR (MH "Noncommunicable Diseases") ) OR (MH "Comorbidity") OR TI ( (("long term" or chronic) n3 (disease# or illness* or condition# or disorder#)) ) OR AB ( (("long term" or chronic) n3 (disease# or illness* or condition# or disorder#)) ) OR TI (co-morbid* or comorbid* ) OR AB (co-morbid* or comorbid* ) OR TI (multi-morbid* or multimorbid*) OR AB (multi-morbid* or multimorbid*) 285,637

S20 S18 OR S19 316,783

S19 TX "common mental disorder*" OR TX emotional trauma OR TX psychological trauma 6,770

S18 TX (MH "Depression+") OR TI ( depression or depressive or depressed or dysthymia ) OR TI ( mood or moods or "mental health" ) OR AB ( depression or depressive or depressed or dysthymia ) OR AB ( mood or moods or "mental health" ) 312,733

S17 S1 OR S2 OR S3 OR S4 OR S5 OR S6 OR S7 OR S8 OR S9 OR S10 OR S11 OR S12 OR S13 OR S14 OR S15 OR S16 561,906

S16 (MH "Health Promotion") OR TI ( health n3 (educat* or promot*) ) OR AB ( health n3 (educat* or promot*) ) 138,440

S15 (MH "Combined Modality Therapy") OR TX ( multimodal or multi-modal or "combined modal*" ) 43,342

S14 TX ( (stress n1 (inoculation or manag* or reduc* or resist*)) ) OR TX (anxiety n3 manage*) OR TX ( "acceptance and commitment" ) 24,317

S13 TX "positive psychology" OR TX "third wave" OR TX ( refram* or re-frame* or reapprais* ) 8,791

S12 TI relaxation OR AB relaxation or TI ( mindful or mindfulness ) OR TI ( counsel#ing or coaching ) or AB ( mindful or mindfulness ) OR AB ( counsel#ing or coaching ) 81,921

S11 TI ( (psychotherap* or psycho-therap*) ) OR TI ( (psycho* or cognit* or CBT) n3 (intervention* or program* or therap* or restructur*) ) OR TI ( "brief therapy" or "brief therapies" ) or AB ( (psychotherap* or psycho-therap*) ) OR AB ( (psycho* or cognit* or CBT) n3 (intervention* or program* or therap* or restructur*) ) OR AB ( "brief therapy" or "brief therapies" ) 57,209

S10 (MH "Stress, Psychological+/TH") 3,015

S9 (MH "Counseling+") 41,340

S8 (MH "Psychotherapy+") 215,438

S7 TX (activit* n2 schedul*) 539

S6 TX reinforce or reinforcer or reinforcement or reinforcements or re-inforcement or re-inforcements 20,111

S5 TX ( psychoeducation or psychoeducational or psycho-education or psycho-educational ) OR TX functional analysis OR TX ( (gain* or reapprais*) n2 focus* ) 15,795

S4 TX ( (pleas* or enjoy* or reward*) n4 (activit* or event?) ) OR TX ( (operant or instrumental) n1(conditioning or learning) ) OR TX ( "positive interaction*" or "avoida* coping" or contingency or contingencies) 9,443

S3 TX behavio* and ("self evaluat*" or "self monitor*") 2,872

S2 TI (behavio* n3 (train* or treatment or contracting or modification or modify* or intervention* or program* or therap*)) or AB (behavio* n3 (train* or treatment or contracting or modification or modify* or intervention* or program* or therap*)) 46,372

S1 TX (behavio* n1 activat*) or BATD 1,022

CINAHL (EBSCOhost) 1981+ Search 2

Date searched: 05/05/2022

Records found: 94

# Query Results

S42 S17 AND S28 AND S40 AND S41 94

S41 ( (MH "Bangladesh") OR (MH "Pakistan") ) OR TI ( bangladesh* or pakistan* ) OR AB ( bangladesh* or pakistan* ) OR AF ( bangladesh* or pakistan* ) 20,555

S40 S29 OR S30 OR S31 OR S32 OR S33 OR S34 OR S35 OR S36 OR S37 OR S38 OR S39 1,093,111

S39 TI ( ((mood or paranoid or psychotic or neurologic* or nervous or nervous system or eating) n1 (disorder or disorders or disease or diseases or illness or illnesses or symptom or symptoms)) ) OR AB ( ((mood or paranoid or psychotic or neurologic* or nervous or nervous system or eating) n1 (disorder or disorders or disease or diseases or illness or illnesses or symptom or symptoms)) ) 43,740

S38 TI ( ((stress or cognitive or cognition or personality or "impulse control") n1 (disorder or disorders or disease or diseases or illness or illnesses or symptom or symptoms)) ) OR AB ( ((stress or cognitive or cognition or personality or "impulse control") n1 (disorder or disorders or disease or diseases or illness or illnesses or symptom or symptoms)) ) 36,045

S37 TX ( (("substance abuse" or "substance use" or "drug abuse" or "drug use") n2 disorder#) ) OR TX personality n2 disorder# OR TX ( (sleep n2 (disorder? or syndrome#)) ) 89,447

S36 TX ( ((post-trauma* or posttrauma*) n3 (stress* or disorder or disorders)) ) OR TI ( ("stress syndrome#" or "distress syndrome#" or "pain disorder#" or dementia or alzheimer*) ) OR AB ( ("stress syndrome#" or "distress syndrome#" or "pain disorder#" or dementia or alzheimer*) ) 128,072

S35 TX ( (PTSD or "psychological trauma" or psychotrauma* or "combat disorder#" or "war disorder#") ) OR TX ( ((bipolar or behavio#ral or obsessive or compulsive or panic or mood or delusional) n1 (disorder# or illness* or disease#)) ) OR TX ( (trichotillomani* or OCD or "obsess*-compulsi*" or GAD or "stress reaction#" or "acute stress" or neurosis or neuroses or neurotic) ) 61,248

S34 TX ( dissociative or dissociation ) OR TX ( (somat* or hysteria or hysterical or briquet or multisomat* or MUPs or medically unexplained) ) OR TX ( (affective* n1 (disorder or disorders or disease or diseases or illness or illnesses or symptom or symptoms)) ) 68,845

S33 TX (somat* or hysteria or hysterical or briquet or multisomat* or MUPs or medically unexplained) 32,981

S32 TX schizo* or akathisia or acathisia or neuroleptic-induc* 41,537

S31 TI ( mental or mentally or psychiatr* or psycho* or depression or depressive or depressed or MDD or anxi* or phobia or phobic or agoraphobi* or dysthymi* or ADNOS ) OR AB ( mental or mentally or psychiatr* or psycho* or depression or depressive or depressed or MDD or anxi* or phobia or phobic or agoraphobi* or dysthymi* or ADNOS ) 616,138

S30 (MH "Behavioral Symptoms") 2,975

S29 (MH "Mental Disorders+") 620,324

S28 S18 OR S19 OR S20 OR S21 OR S22 OR S23 OR S24 OR S25 OR S26 OR S27 2,054,194

S27 TX (transi* n3 isch?em* n3 attack?) OR TX ( ((noncommunicable or non-communicable or non-infectious or non infectious) n3 (disease? or illness* or condition? or disorder?)) ) or TI (cancer* or carcinoma? or tumor? or tumour? or neoplasm? or malignan*) or AB (cancer* or carcinoma? or tumor? or tumour? or neoplasm? or malignan*) 646,255

S26 TX ( ((brain? or cerebr* or cerebell* or intracran* or intracerebral) n2 (isch?emi* or infarct* or thrombo* or emboli* or occlus*)) ) OR TX ( ((brain? or cerebr* or cerebell* or intracerebral or intracranial or subarachnoid) n2 (h?emorrhage? or h?ematoma* or bleed*)) ) 17,791

S25 TX ( ((long-term or Chronic*) n2 lung* n5 (condition* or disease* or symptom* or problem* or failure*)) ) OR TX ( (respiratory n2 (condition* or disease* or symptom* or problem*)) ) AND TX ( (stroke or poststroke or post-stroke or cerebrovasc* or brain vasc* or cerebral vasc* or apoplex*) ) 5,204

S24 TX ( ((long-term or chronic*) n2 (airway* or pulmonar* or airflow* or lung*) n2 Obstruct*) ) OR TX (pulmonar* n2 emphysem*) OR TX ( ((long-term or chronic*) n5 (bronchitis or respirat*)) ) 33,285

S23 TI (diabet*) or AB (diabet*) 228,014

S22 TI ( Aneurysm* or Atherosclerosis or Cardio* or endocard* or pericard* ) OR TI ( (heart or cardiac or valvular or coronary or arterial or vascular or hypertension) ) OR AB ( Aneurysm* or Atherosclerosis or Cardio* or endocard* or pericard* ) OR AB ( (heart or cardiac or valvular or coronary or arterial or vascular or hypertension) ) 670,221

S21 (MH "Neoplasms+") OR (MH "Diabetes Mellitus+") 796,252

S20 (MH "Stroke+") OR (MH "Pulmonary Disease, Chronic Obstructive+") OR (MH "Cardiovascular Diseases+") 664,699

S19 (MH "Diabetes Mellitus+") 182,209

S18 ( (MH "Chronic Disease") OR (MH "Noncommunicable Diseases") ) OR (MH "Comorbidity") OR TI ( (("long term" or chronic) n3 (disease# or illness* or condition# or disorder#)) ) OR AB ( (("long term" or chronic) n3 (disease# or illness* or condition# or disorder#)) ) OR TI (co-morbid* or comorbid* ) OR AB (co-morbid* or comorbid* ) OR TI (multi-morbid* or multimorbid*) OR AB (multi-morbid* or multimorbid*) 285,637

S17 S1 OR S2 OR S3 OR S4 OR S5 OR S6 OR S7 OR S8 OR S9 OR S10 OR S11 OR S12 OR S13 OR S14 OR S15 OR S16 561,906

S16 (MH "Health Promotion") OR TI ( health n3 (educat* or promot*) ) OR AB ( health n3 (educat* or promot*) ) 138,440

S15 (MH "Combined Modality Therapy") OR TX ( multimodal or multi-modal or "combined modal*" ) 43,342

S14 TX ( (stress n1 (inoculation or manag* or reduc* or resist*)) ) OR TX (anxiety n3 manage*) OR TX ( "acceptance and commitment" ) 24,317

S13 TX "positive psychology" OR TX "third wave" OR TX ( refram* or re-frame* or reapprais* ) 8,791

S12 TI relaxation OR AB relaxation or TI ( mindful or mindfulness ) OR TI ( counsel#ing or coaching ) or AB ( mindful or mindfulness ) OR AB ( counsel#ing or coaching ) 81,921

S11 TI ( (psychotherap* or psycho-therap*) ) OR TI ( (psycho* or cognit* or CBT) n3 (intervention* or program* or therap* or restructur*) ) OR TI ( "brief therapy" or "brief therapies" ) or AB ( (psychotherap* or psycho-therap*) ) OR AB ( (psycho* or cognit* or CBT) n3 (intervention* or program* or therap* or restructur*) ) OR AB ( "brief therapy" or "brief therapies" ) 57,209

S10 (MH "Stress, Psychological+/TH") 3,015

S9 (MH "Counseling+") 41,340

S8 (MH "Psychotherapy+") 215,438

S7 TX (activit* n2 schedul*) 539

S6 TX reinforce or reinforcer or reinforcement or reinforcements or re-inforcement or re-inforcements 20,111

S5 TX ( psychoeducation or psychoeducational or psycho-education or psycho-educational ) OR TX functional analysis OR TX ( (gain* or reapprais*) n2 focus* ) 15,795

S4 TX ( (pleas* or enjoy* or reward*) n4 (activit* or event?) ) OR TX ( (operant or instrumental) n1(conditioning or learning) ) OR TX ( "positive interaction*" or "avoida* coping" or contingency or contingencies) 9,443

S3 TX behavio* and ("self evaluat*" or "self monitor*") 2,872

S2 TI (behavio* n3 (train* or treatment or contracting or modification or modify* or intervention* or program* or therap*)) or AB (behavio* n3 (train* or treatment or contracting or modification or modify* or intervention* or program* or therap*)) 46,372

S1 TX (behavio* n1 activat*) or BATD 1,022

Embase Classic+Embase (Ovid) 1947 to 2022 May 04 Search 1

Date searched: 05/05/2022

Records found: 905

1 ((behavio* adj1 activat*) or BATD).tw,kw. (3216)

2 behavio*.mp. and (self adj (evaluat* or monitor*)).tw,kw. (5792)

3 (behavio* adj (train* or treatment)).tw,kw. (8642)

4 (behavio* adj2 (contracting or modification or modify*)).tw,kw. (9607)

5 reinforc*.ti,kw. (24792)

6 (reinforce or reinforcer or reinforcement or reinforcements or re-inforcement or re-inforcements).ab. /freq=2 (14878)

7 (reinforc* adj3 (behavio* or environment* or experience*)).tw,kw. (5695)

8 (reinforc* adj1 (positive or contingent)).tw,kw. (3635)

9 (activit* adj2 schedul*).tw,kw. (939)

10 ((pleas* or enjoy* or reward*) adj4 (activit* or event?)).tw,kw. (6122)

11 ((operant or instrumental) adj (conditioning or learning)).tw,kw. (4221)

12 (positive interaction* or avoida* coping or environmental contingenc* or contingency management).tw,kw. (6889)

13 functional analysis.tw,kw. (34117)

14 ((gain? or reapprais*) adj2 focus*).tw,kw. (221)

15 ((psychoeducat* or psycho-educat*) and (behavi* or coping or self manag*)).ti,ab,kw. (5323)

16 or/1-15 [Behavioural Activation] (119440)

17 exp psychotherapy/ (297146)

18 mental stress/th [Therapy] (1462)

19 (psychotherap* or psycho-therap*).tw,kw. (74244)

20 (behav$ adj3 (intervention$ or program$ or therap$)).tw,kw. (79097)

21 ((cognit$ or CBT) adj3 (intervention$ or program$ or therap$)).tw,kw. (50655)

22 (psycho$ adj3 (intervention$ or program$ or therap$)).tw,kw. (64037)

23 brief therap*.tw,kw. (699)

24 relaxation.tw,kw. (146754)

25 mindful$.tw,kw. (17975)

26 (counsel?ing or coaching).tw,kw. (167548)

27 (third wave adj (psycho$ or therap$)).tw,kw. (66)

28 cognit$ restructur$.tw,kw. (1519)

29 positive psychology.tw,kw. (1630)

30 (refram$ or re-frame$ or reapprais$).tw,kw. (17582)

31 (stress adj1 (inoculation or manag$ or reduc$ or resist$)).tw,kw. (30522)

32 (anxiety adj3 manage$).tw,kw. (2794)

33 "acceptance and commitment ".tw,kw. (1862)

34 exp counseling/ (191152)

35 (multimodal or multi-modal or combined modal$).tw,kw. (75514)

36 exp health promotion/ (109180)

37 (health adj3 (educat$ or promot$)).tw,kw. (151915)

38 or/17-37 [Psychological Interventions] (1075615)

39 16 or 38 [BA or Psychological Interventions] (1171062)

40 *comorbidity/ (21074)

41 *multiple chronic conditions/ (2675)

42 *chronic disease/ (31686)

43 (co-morbid* or comorbid*).ti,ab,kw. (405204)

44 (multi-morbid* or multimorbid*).ti,ab,kw. (10886)

45 (("long term" or chronic) adj3 (disease? or illness* or condition? or disorder?)).tw,kw. (611376)

46 exp *diabetes mellitus/ (577860)

47 exp *cardiovascular disease/ (2960885)

48 *chronic obstructive lung disease/ (77053)

49 exp *cerebrovascular accident/ (103584)

50 diabet*.tw,kw. (1119165)

51 (Aneurysm* or Atherosclerosis or Cardio* or endocard* or pericard*).tw,kw. (1766521)

52 (heart or cardiac or valvular or coronary or arterial or vascular or hypertension).tw,kw. (3697713)

53 ((long-term or chronic*) adj2 (airway* or pulmonar* or airflow* or lung*) adj2 Obstruct*).tw,kw. (89825)

54 (pulmonar* adj2 emphysem*).tw,kw. (7715)

55 ((long-term or chronic*) adj5 (bronchitis or respirat*)).tw,kw. (47048)

56 ((long-term or Chronic*) adj2 lung* adj5 (condition* or disease* or symptom* or problem* or failure*)).tw,kw. (24195)

57 (respiratory adj2 (condition* or disease* or symptom* or problem*)).tw,kw. (101045)

58 (stroke or poststroke or post-stroke or cerebrovasc* or brain vasc* or cerebral vasc* or apoplex*).tw,kw. (543215)

59 ((brain? or cerebr* or cerebell* or intracran* or intracerebral) adj2 (isch?emi* or infarct* or thrombo* or emboli* or occlus*)).tw,kw. (136915)

60 ((brain? or cerebr* or cerebell* or intracerebral or intracranial or subarachnoid) adj2 (h?emorrhage? or h?ematoma* or bleed*)).tw,kw. (106540)

61 (transi* adj3 isch?em* adj3 attack?).tw,kw. (25770)

62 exp *neoplasm/ (4003350)

63 (cancer* or carcinoma? or tumor? or tumour? or neoplasm? or malignan*).tw,kw. (5103414)

64 ((noncommunicable or non-communicable or non-infectious or non infectious) adj3 (disease? or illness* or condition? or disorder?)).tw,kw. (19430)

65 *non communicable disease/ (3717)

66 or/40-65 [NCDs chronic disease] (12345130)

67 exp *depression/ (242895)

68 (depressi* or depressed).tw,kw. (709842)

69 dysthymi*.tw,kw. (4460)

70 (mood? or mental health).tw,kw. (352087)

71 ((emotion* or psychological) adj (trauma* or distress*)).tw,kw. (44737)

72 "common mental disorder*".tw,kw. (3838)

73 or/67-72 [Depression] (1008746)

74 ((Indian or Indians) not ("west indian*" or "american indian*")).ti,ab,in,ad,kw. (194569)

75 india.ti,ab,in,ad,kw. (1021022)

76 ("south asia*" or bangladesh* or pakistan*).ti,ab,in,ad,kw. (164638)

77 (Bhutan* or afghanistan* or maldive* or nepal* or "sri lanka*").ti,ab,in,ad,kw. (55693)

78 exp south asia/ (256403)

79 or/74-78 [S Asia] (1287402)

80 39 and 66 and 73 and 79 [Psychol Interventions and Depression and S Asia and NCDs] (905)

Embase Classic+Embase (Ovid) 1947 to 2022 May 04 Search 2

Date searched: 05/05/2022

Records found: 310

1 ((behavio* adj1 activat*) or BATD).tw,kw. (3216)

2 behavio*.mp. and (self adj (evaluat* or monitor*)).tw,kw. (5792)

3 (behavio* adj (train* or treatment)).tw,kw. (8642)

4 (behavio* adj2 (contracting or modification or modify*)).tw,kw. (9607)

5 reinforc*.ti,kw. (24792)

6 (reinforce or reinforcer or reinforcement or reinforcements or re-inforcement or re-inforcements).ab. /freq=2 (14878)

7 (reinforc* adj3 (behavio* or environment* or experience*)).tw,kw. (5695)

8 (reinforc* adj1 (positive or contingent)).tw,kw. (3635)

9 (activit* adj2 schedul*).tw,kw. (939)

10 ((pleas* or enjoy* or reward*) adj4 (activit* or event?)).tw,kw. (6122)

11 ((operant or instrumental) adj (conditioning or learning)).tw,kw. (4221)

12 (positive interaction* or avoida* coping or environmental contingenc* or contingency management).tw,kw. (6889)

13 functional analysis.tw,kw. (34117)

14 ((gain? or reapprais*) adj2 focus*).tw,kw. (221)

15 ((psychoeducat* or psycho-educat*) and (behavi* or coping or self manag*)).ti,ab,kw. (5323)

16 or/1-15 [Behavioural Activation] (119440)

17 exp psychotherapy/ (297146)

18 mental stress/th [Therapy] (1462)

19 (psychotherap* or psycho-therap*).tw,kw. (74244)

20 (behav$ adj3 (intervention$ or program$ or therap$)).tw,kw. (79097)

21 ((cognit$ or CBT) adj3 (intervention$ or program$ or therap$)).tw,kw. (50655)

22 (psycho$ adj3 (intervention$ or program$ or therap$)).tw,kw. (64037)

23 brief therap*.tw,kw. (699)

24 relaxation.tw,kw. (146754)

25 mindful$.tw,kw. (17975)

26 (counsel?ing or coaching).tw,kw. (167548)

27 (third wave adj (psycho$ or therap$)).tw,kw. (66)

28 cognit$ restructur$.tw,kw. (1519)

29 positive psychology.tw,kw. (1630)

30 (refram$ or re-frame$ or reapprais$).tw,kw. (17582)

31 (stress adj1 (inoculation or manag$ or reduc$ or resist$)).tw,kw. (30522)

32 (anxiety adj3 manage$).tw,kw. (2794)

33 "acceptance and commitment ".tw,kw. (1862)

34 exp counseling/ (191152)

35 (multimodal or multi-modal or combined modal$).tw,kw. (75514)

36 exp health promotion/ (109180)

37 (health adj3 (educat$ or promot$)).tw,kw. (151915)

38 or/17-37 [Psychological Interventions] (1075615)

39 16 or 38 [BA or Psychological Interventions] (1171062)

40 exp mental disease/ (2591520)

41 (mental or mentally or psychiatr* or psycho* or depressi* or depressed or MDD or anxi* or phobia or phobic or agoraphobi* or dysthymi* or ADNOS).tw,kw. (2213721)

42 (schizo* or hebephrenic* or oligophreni* or akathisi* or acathisi* or neuroleptic-induc*).tw,kw. (219515)

43 (somatoform or somatiz* or somatis* or hysteri* or briquet or multisomat* or multi somat* or MUPs or medically unexplained).tw,kw. (21582)

44 ((dissociative adj3 (disorder* or reaction*)) or dissociation).tw,kw. (128575)

45 (affective* adj (disorder? or disease? or illness* or symptom?)).tw,kw. (27812)

46 (PTSD or psychological trauma or psychotrauma* or combat disorder? or war disorder?).tw,kw. (41705)

47 ((post-trauma* or posttrauma*) adj3 (stress* or disorder?)).tw,kw. (49025)

48 ((stress or cognitive or cognition or personality or impulse control or mood or paranoid or psychotic or neurologic* or nervous or nervous system or eating) adj (disorder? or illness* or disease?)).tw,kw. (247183)

49 ((bipolar or behavio?ral or obsessive or compulsive or panic or mood or delusional) adj (disorder? or illness* or disease?)).tw,kw. (112954)

50 (trichotillomani* or OCD or obsess*-compulsi* or GAD or stress reaction? or acute stress or neuros#s or neurotic).tw,kw. (85642)

51 (stress syndrome? or distress syndrome? or pain disorder? or dementia or alzheimer? or epilepsy).tw,kw. (575611)

52 ((substance abuse or "substance use" or drug abuse or "drug use") adj2 disorder?).tw,kw. (26648)

53 (personality adj2 disorder?).tw,kw. (29782)

54 (sleep? adj2 (disorder? or syndrome?)).tw,kw. (47828)

55 or/40-54 [MENTAL DISORDERS] (4134046)

56 *comorbidity/ (21074)

57 *multiple chronic conditions/ (2675)

58 *chronic disease/ (31686)

59 (co-morbid* or comorbid*).ti,ab,kw. (405204)

60 (multi-morbid* or multimorbid*).ti,ab,kw. (10886)

61 (("long term" or chronic) adj3 (disease? or illness* or condition? or disorder?)).tw,kw. (611376)

62 exp *diabetes mellitus/ (577860)

63 exp *cardiovascular disease/ (2960885)

64 *chronic obstructive lung disease/ (77053)

65 exp *cerebrovascular accident/ (103584)

66 diabet*.tw,kw. (1119165)

67 (Aneurysm* or Atherosclerosis or Cardio* or endocard* or pericard*).tw,kw. (1766521)

68 (heart or cardiac or valvular or coronary or arterial or vascular or hypertension).tw,kw. (3697713)

69 ((long-term or chronic*) adj2 (airway* or pulmonar* or airflow* or lung*) adj2 Obstruct*).tw,kw. (89825)

70 (pulmonar* adj2 emphysem*).tw,kw. (7715)

71 ((long-term or chronic*) adj5 (bronchitis or respirat*)).tw,kw. (47048)

72 ((long-term or Chronic*) adj2 lung* adj5 (condition* or disease* or symptom* or problem* or failure*)).tw,kw. (24195)

73 (respiratory adj2 (condition* or disease* or symptom* or problem*)).tw,kw. (101045)

74 (stroke or poststroke or post-stroke or cerebrovasc* or brain vasc* or cerebral vasc* or apoplex*).tw,kw. (543215)

75 ((brain? or cerebr* or cerebell* or intracran* or intracerebral) adj2 (isch?emi* or infarct* or thrombo* or emboli* or occlus*)).tw,kw. (136915)

76 ((brain? or cerebr* or cerebell* or intracerebral or intracranial or subarachnoid) adj2 (h?emorrhage? or h?ematoma* or bleed*)).tw,kw. (106540)

77 (transi* adj3 isch?em* adj3 attack?).tw,kw. (25770)

78 exp *neoplasm/ (4003350)

79 (cancer* or carcinoma? or tumor? or tumour? or neoplasm? or malignan*).tw,kw. (5103414)

80 ((noncommunicable or non-communicable or non-infectious or non infectious) adj3 (disease? or illness* or condition? or disorder?)).tw,kw. (19430)

81 *non communicable disease/ (3717)

82 or/56-81 [NCDs chronic disease] (12345130)

83 (bangladesh* or pakistan*).ti,ab,in,ad,kw. (151244)

84 bangladesh/ or exp pakistan/ (52166)

85 83 or 84 (156058)

86 39 and 55 and 82 and 85 (310)

Global Health (Ovid) 1910 to 2022 Week 17 Search 1:

Date searched: 05/05/2022

Records found: 155

1 ((behavio* adj1 activat*) or BATD).tw,id. 155

2 behavio*.mp. and (self adj (evaluat* or monitor*)).tw,id. 848

3 (behavio* adj (train* or treatment)).tw,id. 530

4 (behavio* adj2 (contracting or modification or modify*)).tw,id. 3274

5 reinforc*.ti,id. 958

6 (reinforce or reinforcer or reinforcement or reinforcements or re-inforcement or re-inforcements).ab. /freq=2 692

7 (reinforc* adj3 (behavio* or environment* or experience*)).tw,id. 447

8 (reinforc* adj1 (positive or contingent)).tw,id. 244

9 (activit* adj2 schedul*).tw,id. 92

10 ((pleas* or enjoy* or reward*) adj4 (activit* or event?)).tw,id. 574

11 ((operant or instrumental) adj (conditioning or learning)).tw,id. 134

12 (positive interaction* or avoida* coping or environmental contingenc* or contingency management).tw,id. 813

13 functional analysis.tw,id. 2052

14 ((gain? or reapprais*) adj2 focus*).tw,id. 32

15 ((psychoeducat* or psycho-educat*) and (behavi* or coping or self manag*)).ti,ab,id. 281

16 or/1-15 [Behavioural Activation] 10423

17 exp psychotherapy/ 4182

18 exp counselling/ 12781

19 (psychotherap* or psycho-therap*).tw,id. 2728

20 (behav$ adj3 (intervention$ or program$ or therap$)).tw,id. 12594

21 ((cognit$ or CBT) adj3 (intervention$ or program$ or therap$)).tw,id. 2594

22 (psycho$ adj3 (intervention$ or program$ or therap$)).tw,id. 4395

23 brief therap*.tw,id. 14

24 relaxation.tw,id. 7061

25 mindful$.tw,id. 1339

26 (counsel?ing or coaching).tw,id. 25885

27 (third wave adj (psycho$ or therap$)).tw,id. 2

28 cognit$ restructur$.tw,id. 61

29 positive psychology.tw,id. 102

30 (refram$ or re-frame$ or reapprais$).tw,id. 1612

31 (stress adj1 (inoculation or manag$ or reduc$ or resist$)).tw,id. 4325

32 (anxiety adj3 manage$).tw,id. 171

33 "acceptance and commitment ".tw,id. 137

34 (multimodal or multi-modal or combined modal$).tw,id. 8901

35 health promotion/ 29041

36 (health adj3 (educat$ or promot$)).tw,id. 84241

37 or/17-36 [Psychological Interventions] 144279

38 16 or 37 [BA or Psychological Interventions] 151605

39 (co-morbid* or comorbid*).ti,ab,id. 36198

40 (multi-morbid* or multimorbid*).ti,ab,id. 1366

41 (("long term" or chronic) adj3 (disease? or illness* or condition? or disorder?)).tw,id. 88504

42 diabetes/ or diabetes mellitus/ 104872

43 exp cardiovascular diseases/ 149389

44 exp stroke/ 14986

45 chronic obstructive pulmonary disease/ 8109

46 exp neoplasms/ 277956

47 diabet*.tw,id. 163553

48 (Aneurysm* or Atherosclerosis or Cardio* or endocard* or pericard*).tw,id. 165582

49 (heart or cardiac or valvular or coronary or arterial or vascular or hypertension).tw,id. 245001

50 ((long-term or chronic*) adj2 (airway* or pulmonar* or airflow* or lung*) adj2 Obstruct*).tw,id. 10404

51 (pulmonar* adj2 emphysem*).tw,id. 1929

52 ((long-term or chronic*) adj5 (bronchitis or respirat*)).tw,id. 6543

53 ((long-term or Chronic*) adj2 lung* adj5 (condition* or disease* or symptom* or problem* or failure*)).tw,id. 2339

54 (respiratory adj2 (condition* or disease* or symptom* or problem*)).tw,id. 178233

55 (stroke or poststroke or post-stroke or cerebrovasc* or brain vasc* or cerebral vasc* or apoplex*).tw,id. 30227

56 ((brain? or cerebr* or cerebell* or intracran* or intracerebral) adj2 (isch?emi* or infarct* or thrombo* or emboli* or occlus*)).tw,id. 7515

57 ((brain? or cerebr* or cerebell* or intracerebral or intracranial or subarachnoid) adj2 (h?emorrhage? or h?ematoma* or bleed*)).tw,id. 4235

58 (transi* adj3 isch?em* adj3 attack?).tw,id. 769

59 (cancer* or carcinoma? or tumor? or tumour? or neoplasm? or malignan*).tw,id. 376260

60 ((noncommunicable or non-communicable or non-infectious or non infectious) adj3 (disease? or illness* or condition? or disorder?)).tw,id. 10435

61 or/39-60 [NCDs chronic disease] 973743

62 depression/ 29697

63 (depressi* or depressed).tw,id. 63693

64 dysthymi*.tw,id. 226

65 (mood? or mental health).tw,id. 50008

66 ((emotion* or psychological) adj (trauma* or distress*)).tw,id. 5005

67 "common mental disorder*".tw,id. 926

68 or/62-67 [Depression] 99867

69 ((Indian or Indians) not ("west indian*" or "american indian*")).ti,ab,in,gl. 53603

70 india.ti,ab,in,gl. 261341

71 ("south asia*" or bangladesh* or pakistan*).ti,ab,in,gl. 56968

72 (Bhutan* or afghanistan* or maldive* or nepal* or "sri lanka*").ti,ab,in,gl. 23350

73 exp south asia/ or afghanistan/ or maldives/ 190937

74 or/69-73 [S Asia] 342390

75 38 and 61 and 68 and 74 [Psychol Interventions and Depression and S Asia and NCDs] 155

Global Health (Ovid) 1910 to 2022 Week 17 Search 2:

Date searched: 05/05/2022

Records found: 66

Global Health <1910 to 2022 Week 17>

1 ((behavio* adj1 activat*) or BATD).tw,id. 155

2 behavio*.mp. and (self adj (evaluat* or monitor*)).tw,id. 848

3 (behavio* adj (train* or treatment)).tw,id. 530

4 (behavio* adj2 (contracting or modification or modify*)).tw,id. 3274

5 reinforc*.ti,id. 958

6 (reinforce or reinforcer or reinforcement or reinforcements or re-inforcement or re-inforcements).ab. /freq=2 692

7 (reinforc* adj3 (behavio* or environment* or experience*)).tw,id. 447

8 (reinforc* adj1 (positive or contingent)).tw,id. 244

9 (activit* adj2 schedul*).tw,id. 92

10 ((pleas* or enjoy* or reward*) adj4 (activit* or event?)).tw,id. 574

11 ((operant or instrumental) adj (conditioning or learning)).tw,id. 134

12 (positive interaction* or avoida* coping or environmental contingenc* or contingency management).tw,id. 813

13 functional analysis.tw,id. 2052

14 ((gain? or reapprais*) adj2 focus*).tw,id. 32

15 ((psychoeducat* or psycho-educat*) and (behavi* or coping or self manag*)).ti,ab,id. 281

16 or/1-15 [Behavioural Activation] 10423

17 exp psychotherapy/ 4182

18 exp counselling/ 12781

19 (psychotherap* or psycho-therap*).tw,id. 2728

20 (behav$ adj3 (intervention$ or program$ or therap$)).tw,id. 12594

21 ((cognit$ or CBT) adj3 (intervention$ or program$ or therap$)).tw,id. 2594

22 (psycho$ adj3 (intervention$ or program$ or therap$)).tw,id. 4395

23 brief therap*.tw,id. 14

24 relaxation.tw,id. 7061

25 mindful$.tw,id. 1339

26 (counsel?ing or coaching).tw,id. 25885

27 (third wave adj (psycho$ or therap$)).tw,id. 2

28 cognit$ restructur$.tw,id. 61

29 positive psychology.tw,id. 102

30 (refram$ or re-frame$ or reapprais$).tw,id. 1612

31 (stress adj1 (inoculation or manag$ or reduc$ or resist$)).tw,id. 4325

32 (anxiety adj3 manage$).tw,id. 171

33 "acceptance and commitment ".tw,id. 137

34 (multimodal or multi-modal or combined modal$).tw,id. 8901

35 health promotion/ 29041

36 (health adj3 (educat$ or promot$)).tw,id. 84241

37 or/17-36 [Psychological Interventions] 144279

38 16 or 37 [BA or Psychological Interventions] 151605

39 (co-morbid* or comorbid*).ti,ab,id. 36198

40 (multi-morbid* or multimorbid*).ti,ab,id. 1366

41 (("long term" or chronic) adj3 (disease? or illness* or condition? or disorder?)).tw,id. 88504

42 diabetes/ or diabetes mellitus/ 104872

43 exp cardiovascular diseases/ 149389

44 exp stroke/ 14986

45 chronic obstructive pulmonary disease/ 8109

46 exp neoplasms/ 277956

47 diabet*.tw,id. 163553

48 (Aneurysm* or Atherosclerosis or Cardio* or endocard* or pericard*).tw,id. 165582

49 (heart or cardiac or valvular or coronary or arterial or vascular or hypertension).tw,id. 245001

50 ((long-term or chronic*) adj2 (airway* or pulmonar* or airflow* or lung*) adj2 Obstruct*).tw,id. 10404

51 (pulmonar* adj2 emphysem*).tw,id. 1929

52 ((long-term or chronic*) adj5 (bronchitis or respirat*)).tw,id. 6543

53 ((long-term or Chronic*) adj2 lung* adj5 (condition* or disease* or symptom* or problem* or failure*)).tw,id. 2339

54 (respiratory adj2 (condition* or disease* or symptom* or problem*)).tw,id. 178233

55 (stroke or poststroke or post-stroke or cerebrovasc* or brain vasc* or cerebral vasc* or apoplex*).tw,id. 30227

56 ((brain? or cerebr* or cerebell* or intracran* or intracerebral) adj2 (isch?emi* or infarct* or thrombo* or emboli* or occlus*)).tw,id. 7515

57 ((brain? or cerebr* or cerebell* or intracerebral or intracranial or subarachnoid) adj2 (h?emorrhage? or h?ematoma* or bleed*)).tw,id. 4235

58 (transi* adj3 isch?em* adj3 attack?).tw,id. 769

59 (cancer* or carcinoma? or tumor? or tumour? or neoplasm? or malignan*).tw,id. 376260

60 ((noncommunicable or non-communicable or non-infectious or non infectious) adj3 (disease? or illness* or condition? or disorder?)).tw,id. 10435

61 or/39-60 [NCDs chronic disease] 973743

62 exp mental disorders/ 93392

63 (mental or mentally or psychiatr* or psycho* or depressi* or depressed or MDD or anxi* or phobia or phobic or agoraphobi* or dysthymi* or ADNOS).tw,id. 223489

64 (schizo* or hebephrenic* or oligophreni* or akathisi* or acathisi* or neuroleptic-induc*).tw,id. 14711

65 (somatoform or somatiz* or somatis* or hysteri* or briquet or multisomat* or multi somat* or MUPs or medically unexplained).tw,id. 1514

66 ((dissociative adj3 (disorder* or reaction*)) or dissociation).tw,id. 4779

67 (affective* adj (disorder? or disease? or illness* or symptom?)).tw,id. 2353

68 (PTSD or psychological trauma or psychotrauma* or combat disorder? or war disorder?).tw,id. 2649

69 ((post-trauma* or posttrauma*) adj3 (stress* or disorder?)).tw,id. 4003

70 ((stress or cognitive or cognition or personality or impulse control or mood or paranoid or psychotic or neurologic* or nervous or nervous system or eating) adj (disorder? or illness* or disease?)).tw,id. 35225

71 ((bipolar or behavio?ral or obsessive or compulsive or panic or mood or delusional) adj (disorder? or illness* or disease?)).tw,id. 5487

72 (trichotillomani* or OCD or obsess*-compulsi* or GAD or stress reaction? or acute stress or neuros#s or neurotic).tw,id. 4418

73 (stress syndrome? or distress syndrome? or pain disorder? or dementia or alzheimer? or epilepsy).tw,id. 35350

74 ((substance abuse or "substance use" or drug abuse or "drug use") adj2 disorder?).tw,id. 3174

75 (personality adj2 disorder?).tw,id. 945

76 (sleep? adj2 (disorder? or syndrome?)).tw,id. 2974

77 or/62-76 [Mental Disorders] 282560

78 (bangladesh* or pakistan*).ti,ab,in,gl. 52548

79 exp bangladesh/ or exp pakistan/ 34553

80 78 or 79 52601

81 38 and 61 and 77 and 80 [Psy Interventions NCDs Mental Disorders_Pakistan Bangladesh] 66

IMEMR Index Medicus for the Eastern Mediterranean (WHO Global Health Index Medicus) Search 1

Date searched: 05/05/2022

Records found: 7

Advanced search mode

Title, subject, abstract: behavio* or psychotherap* or psychoeducation* or psychological*

AND

Title, subject, abstract: chronic or noncommunicable or comorbid* or diabet* or stroke* or cardio* or pulmonary or lung* or cancer* or neoplasm*

AND

Title, subject, abstract: Bhutan* OR afghanistan* OR maldive* OR nepal* OR "sri lanka" OR india* OR bangladesh* OR pakistan* OR "south asia"

AND

Title, subject, abstract: depress* OR dysthymia*

IMEMR Index Medicus for the Eastern Mediterranean (WHO Global Health Index Medicus)

Search 2

Date searched: 05/05/2022

Records found: 14

Advanced search

Title, subject, abstract: behavio* or psychotherap* or psychoeducation* or psychological*

AND

Title, subject, abstract: chronic or noncommunicable or comorbid* or diabet* or stroke* or cardio* or pulmonary or lung* or cancer* or neoplasm*

AND

Title, subject, abstract: bangladesh* OR pakistan*

AND

Title, subject, abstract: mental* OR psychiatr* OR psycho* OR depress* OR anxi* OR phobia OR phobic OR dysthymia* OR schizo* OR somat* OR hysteri* OR briquet OR multisomat* OR affective* OR bipolar OR obsessive OR compulsive OR panic OR mood OR delusional OR neurologic* OR nervous

IMSEAR Index Medicus for the South East Asia Region (WHO Global Health Index Medicus) Search 1

Date searched: 05/05/2022

Records found: 15

Same search strategy as IMEAR

IMSEAR Index Medicus for the South East Asia Region (WHO Global Health Index Medicus) Search 2

Date searched: 05/05/2022

Records found: 4

Same search strategy as IMEAR

Ovid MEDLINE(R) ALL 1946 to May 04, 2022 Search 1

Date searched: 05/05/2022

Records found: 474

1 ((behavio* adj1 activat*) or BATD).tw,kf. (2737)

2 behavio*.mp. and (self adj (evaluat* or monitor*)).tw,kf. (4526)

3 (behavio* adj (train* or treatment)).tw,kf. (5963)

4 (behavio* adj2 (contracting or modification or modify*)).tw,kf. (7463)

5 reinforc*.ti,kf. (25637)

6 (reinforce or reinforcer or reinforcement or reinforcements or re-inforcement or re-inforcements).ab. /freq=2 (13179)

7 (reinforc* adj3 (behavio* or environment* or experience*)).tw,kf. (4569)

8 (reinforc* adj1 (positive or contingent)).tw,kf. (2835)

9 (activit* adj2 schedul*).tw,kf. (648)

10 ((pleas* or enjoy* or reward*) adj4 (activit* or event?)).tw,kf. (4478)

11 ((operant or instrumental) adj (conditioning or learning)).tw,kf. (3612)

12 (positive interaction* or avoida* coping or environmental contingenc* or contingency management).tw,kf. (5634)

13 functional analysis.tw,kf. (27900)

14 ((gain? or reapprais*) adj2 focus*).tw,kf. (200)

15 ((psychoeducat* or psycho-educat*) and (behavi* or coping or self manag*)).ti,ab,kf. (3440)

16 or/1-15 [Behavioural Activation] (100125)

17 exp psychotherapy/ (211760)

18 Stress, Psychological/th (6129)

19 (psychotherap* or psycho-therap*).tw,kf. (51070)

20 (behav$ adj3 (intervention$ or program$ or therap$)).tw,kf. (60028)

21 ((cognit$ or CBT) adj3 (intervention$ or program$ or therap$)).tw,kf. (36754)

22 (psycho$ adj3 (intervention$ or program$ or therap$)).tw,kf. (45008)

23 brief therap*.tw,kf. (429)

24 relaxation.tw,kf. (128391)

25 mindful$.tw,kf. (13833)

26 (counsel?ing or coaching).tw,kf. (116520)

27 (third wave adj (psycho$ or therap$)).tw,kf. (51)

28 cognit$ restructur$.tw,kf. (999)

29 positive psychology.tw,kf. (1585)

30 (refram$ or re-frame$ or reapprais$).tw,kf. (14631)

31 (stress adj1 (inoculation or manag$ or reduc$ or resist$)).tw,kf. (24774)

32 (anxiety adj3 manage$).tw,kf. (1941)

33 "acceptance and commitment ".tw,kf. (1478)

34 Combined Modality Therapy/ (183562)

35 (multimodal or multi-modal or combined modal$).tw,kf. (54871)

36 exp Health promotion/ (83128)

37 (health adj3 (educat$ or promot$)).tw,kf. (134405)

38 or/17-37 [Psychological Interventions Kunzler 2020 et al] (957289)

39 16 or 38 [BA or Psychological Interventions] (1039148)

40 Depression/ or exp depressive disorder/ (243975)

41 (depressi* or depressed or dysthymi*).tw,kf. (505698)

42 (mood? or mental health or ((emotion* or psychological) adj trauma*)).tw,kf. (276212)

43 "common mental disorder*".tw,kf. (3293)

44 40 or 41 or 42 or 43 [Depression - Cochrane Terms] (735086)

45 ((Indian or Indians) not ("west indian*" or "american indian*")).ti,ab,in,kf. (146431)

46 (Bhutan* or afghanistan* or maldive* or nepal* or "sri lanka*" or india).ti,ab,in,kf. (675349)

47 ("south asia*" or bangladesh* or pakistan*).ti,ab,in,kf. (111614)

48 asia, western/ or bangladesh/ or bhutan/ or exp india/ or afghanistan/ or nepal/ or pakistan/ or sri lanka/ or exp Indian Ocean Islands/ (174633)

49 or/45-48 [S Asia] (845339)

50 comorbidity/ or multimorbidity/ (123823)

51 (co-morbid* or comorbid*).ti,ab,kf. (228412)

52 (multi-morbid* or multimorbid*).ti,ab,kf. (8221)

53 Chronic Disease/ (275383)

54 (("long term" or chronic) adj3 (disease? or illness* or condition? or disorder?)).tw,kf. (417992)

55 exp Diabetes Mellitus/ (478844)

56 exp Cardiovascular Diseases/ (2615540)

57 exp Pulmonary Disease, Chronic Obstructive/ (63288)

58 exp Stroke/ (159077)

59 diabet*.tw,kf. (722380)

60 (Aneurysm* or Atherosclerosis or Cardio* or endocard* or pericard*).tw,kf. (1182378)

61 (heart or cardiac or valvular or coronary or arterial or vascular or hypertension).tw,kf. (2561154)

62 ((long-term or chronic*) adj2 (airway* or pulmonar* or airflow* or lung*) adj2 Obstruct*).tw. (61322)

63 (pulmonar* adj2 emphysem*).tw. (4975)

64 ((long-term or chronic*) adj5 (bronchitis or respirat*)).tw,kf. (29639)

65 ((long-term or Chronic*) adj2 lung* adj5 (condition* or disease* or symptom* or problem* or failure*)).tw,kf. (15990)

66 (respiratory adj2 (condition* or disease* or symptom* or problem*)).tw,kf. (71626)

67 (stroke or poststroke or post-stroke or cerebrovasc* or brain vasc* or cerebral vasc* or apoplex*).tw. (333394)

68 ((brain? or cerebr* or cerebell* or intracran* or intracerebral) adj2 (isch?emi* or infarct* or thrombo* or emboli* or occlus*)).tw. (92417)

69 ((brain? or cerebr* or cerebell* or intracerebral or intracranial or subarachnoid) adj2 (h?emorrhage? or h?ematoma* or bleed*)).tw. (69468)

70 (transi* adj3 isch?em* adj3 attack?).tw. (16426)

71 exp neoplasm/ (3682540)

72 (cancer* or carcinoma? or tumor? or tumour? or neoplasm? or malignan*).tw,kf. (3682665)

73 ((noncommunicable or non-communicable or non-infectious or non infectious) adj3 (disease? or illness* or condition? or disorder?)).tw,kf. (16341)

74 Noncommunicable Diseases/ (2406)

75 or/50-74 [NCDs Chronic Conditions] (9554530)

76 39 and 44 and 49 and 75 [Psychol Interventions and Depression and S Asia and NCDs] (474)

Ovid MEDLINE(R) ALL 1946 to May 04, 2022 Search 2

Date searched: 05/05/2022

Records found: 153

1 ((behavio* adj1 activat*) or BATD).tw,kf. (2737)

2 behavio*.mp. and (self adj (evaluat* or monitor*)).tw,kf. (4526)

3 (behavio* adj (train* or treatment)).tw,kf. (5963)

4 (behavio* adj2 (contracting or modification or modify*)).tw,kf. (7463)

5 reinforc*.ti,kf. (25637)

6 (reinforce or reinforcer or reinforcement or reinforcements or re-inforcement or re-inforcements).ab. /freq=2 (13179)

7 (reinforc* adj3 (behavio* or environment* or experience*)).tw,kf. (4569)

8 (reinforc* adj1 (positive or contingent)).tw,kf. (2835)

9 (activit* adj2 schedul*).tw,kf. (648)

10 ((pleas* or enjoy* or reward*) adj4 (activit* or event?)).tw,kf. (4478)

11 ((operant or instrumental) adj (conditioning or learning)).tw,kf. (3612)

12 (positive interaction* or avoida* coping or environmental contingenc* or contingency management).tw,kf. (5634)

13 functional analysis.tw,kf. (27900)

14 ((gain? or reapprais*) adj2 focus*).tw,kf. (200)

15 ((psychoeducat* or psycho-educat*) and (behavi* or coping or self manag*)).ti,ab,kf. (3440)

16 or/1-15 [Behavioural Activation] (100125)

17 exp psychotherapy/ (211760)

18 Stress, Psychological/th (6129)

19 (psychotherap* or psycho-therap*).tw,kf. (51070)

20 (behav$ adj3 (intervention$ or program$ or therap$)).tw,kf. (60028)

21 ((cognit$ or CBT) adj3 (intervention$ or program$ or therap$)).tw,kf. (36754)

22 (psycho$ adj3 (intervention$ or program$ or therap$)).tw,kf. (45008)

23 brief therap*.tw,kf. (429)

24 relaxation.tw,kf. (128391)

25 mindful$.tw,kf. (13833)

26 (counsel?ing or coaching).tw,kf. (116520)

27 (third wave adj (psycho$ or therap$)).tw,kf. (51)

28 cognit$ restructur$.tw,kf. (999)

29 positive psychology.tw,kf. (1585)

30 (refram$ or re-frame$ or reapprais$).tw,kf. (14631)

31 (stress adj1 (inoculation or manag$ or reduc$ or resist$)).tw,kf. (24774)

32 (anxiety adj3 manage$).tw,kf. (1941)

33 "acceptance and commitment ".tw,kf. (1478)

34 Combined Modality Therapy/ (183562)

35 (multimodal or multi-modal or combined modal$).tw,kf. (54871)

36 exp Health promotion/ (83128)

37 (health adj3 (educat$ or promot$)).tw,kf. (134405)

38 or/17-37 [Psychological Interventions Kunzler 2020 et al] (957289)

39 16 or 38 [BA or Psychological Interventions] (1039148)

40 exp Mental Disorders/ (1371189)

41 exp Behavioral Symptoms/ (417823)

42 (mental or mentally or psychiatr* or psycho* or depressi* or depressed or MDD or anxi* or phobia or phobic or agoraphobi* or dysthymi* or ADNOS).tw,kf. (1639887)

43 (schizo* or hebephrenic* or oligophreni* or akathisi* or acathisi* or neuroleptic-induc*).tw,kf. (159485)

44 (somatoform or somatiz* or somatis* or hysteri* or briquet or multisomat* or multi somat* or MUPs or medically unexplained).tw,kf. (15148)

45 ((dissociative adj3 (disorder* or reaction*)) or dissociation).tw,kf. (117917)

46 (affective* adj (disorder? or disease? or illness* or symptom?)).tw,kf. (20900)

47 (PTSD or psychological trauma or psychotrauma* or combat disorder? or war disorder?).tw,kf. (31881)

48 ((post-trauma* or posttrauma*) adj3 (stress* or disorder?)).tw,kf. (41386)

49 ((stress or cognitive or cognition or personality or impulse control or mood or paranoid or psychotic or neurologic* or nervous or nervous system or eating) adj (disorder? or illness* or disease?)).tw,kf. (186502)

50 ((bipolar or behavio?ral or obsessive or compulsive or panic or mood or delusional) adj (disorder? or illness* or disease?)).tw,kf. (80224)

51 (trichotillomani* or OCD or obsess*-compulsi* or GAD or stress reaction? or acute stress or neuros#s or neurotic).tw,kf. (61108)

52 (stress syndrome? or distress syndrome? or pain disorder? or dementia or alzheimer? or epilepsy).tw,kf. (407425)

53 ((substance abuse or "substance use" or drug abuse or "drug use") adj2 disorder?).tw,kf. (21508)

54 (personality adj2 disorder?).tw,kf. (22408)

55 (sleep? adj2 (disorder? or syndrome?)).tw,kf. (30991)

56 or/40-55 [MENTAL DISORDERS] (2976318)

57 comorbidity/ or multimorbidity/ (123823)

58 (co-morbid* or comorbid*).ti,ab,kf. (228412)

59 (multi-morbid* or multimorbid*).ti,ab,kf. (8221)

60 Chronic Disease/ (275383)

61 (("long term" or chronic) adj3 (disease? or illness* or condition? or disorder?)).tw,kf. (417992)

62 exp Diabetes Mellitus/ (478844)

63 exp Cardiovascular Diseases/ (2615540)

64 exp Pulmonary Disease, Chronic Obstructive/ (63288)

65 exp Stroke/ (159077)

66 diabet*.tw,kf. (722380)

67 (Aneurysm* or Atherosclerosis or Cardio* or endocard* or pericard*).tw,kf. (1182378)

68 (heart or cardiac or valvular or coronary or arterial or vascular or hypertension).tw,kf. (2561154)

69 ((long-term or chronic*) adj2 (airway* or pulmonar* or airflow* or lung*) adj2 Obstruct*).tw. (61322)

70 (pulmonar* adj2 emphysem*).tw. (4975)

71 ((long-term or chronic*) adj5 (bronchitis or respirat*)).tw,kf. (29639)

72 ((long-term or Chronic*) adj2 lung* adj5 (condition* or disease* or symptom* or problem* or failure*)).tw,kf. (15990)

73 (respiratory adj2 (condition* or disease* or symptom* or problem*)).tw,kf. (71626)

74 (stroke or poststroke or post-stroke or cerebrovasc* or brain vasc* or cerebral vasc* or apoplex*).tw. (333394)

75 ((brain? or cerebr* or cerebell* or intracran* or intracerebral) adj2 (isch?emi* or infarct* or thrombo* or emboli* or occlus*)).tw. (92417)

76 ((brain? or cerebr* or cerebell* or intracerebral or intracranial or subarachnoid) adj2 (h?emorrhage? or h?ematoma* or bleed*)).tw. (69468)

77 (transi* adj3 isch?em* adj3 attack?).tw. (16426)

78 exp neoplasm/ (3682540)

79 (cancer* or carcinoma? or tumor? or tumour? or neoplasm? or malignan*).tw,kf. (3682665)

80 ((noncommunicable or non-communicable or non-infectious or non infectious) adj3 (disease? or illness* or condition? or disorder?)).tw,kf. (16341)

81 Noncommunicable Diseases/ (2406)

82 or/57-81 [NCDs Chronic disease] (9554530)

83 (bangladesh* or pakistan*).ti,ab,in,kf. (101808)

84 bangladesh/ or pakistan/ (33939)

85 83 or 84 [Pakistan or Bangladesh] (106248)

86 39 and 56 and 82 and 85 [Psychol Interventions and Mental Disorders and NCDs and Pakistan_Bangladesh] (153)

APA PsycInfo (Ovid) 1806 to April Week 4 2022 2022 Search 1

Date searched: 05/05/2022

Records found: 153

1 ((behavio* adj1 activat*) or BATD).tw,id. (2718)

2 behavio*.mp. and (self adj (evaluat* or monitor*)).tw,id. (6689)

3 (behavio* adj (train* or treatment)).tw,id. (9945)

4 (behavio* adj2 (contracting or modification or modify*)).tw,id. (8564)

5 reinforc*.ti,id. (28214)

6 (reinforce or reinforcer or reinforcement or reinforcements or re-inforcement or re-inforcements).ab. /freq=2 (16892)

7 (reinforc* adj3 (behavio* or environment* or experience*)).tw,id. (7292)

8 (reinforc* adj1 (positive or contingent)).tw,id. (4889)

9 (activit* adj2 schedul*).tw,id. (659)

10 ((pleas* or enjoy* or reward*) adj4 (activit* or event?)).tw,id. (4925)

11 ((operant or instrumental) adj (conditioning or learning)).tw,id. (6306)

12 (positive interaction* or avoida* coping or environmental contingenc* or contingency management).tw,id. (6389)

13 functional analysis.tw,id. (3476)

14 ((gain? or reapprais*) adj2 focus*).tw,id. (154)

15 ((psychoeducat* or psycho-educat*) and (behavi* or coping or self manag*)).ti,ab,id. (5599)

16 behavioral activation system/ (569)

17 or/1-16 [Behavioural Activation] (87581)

18 exp psychotherapy/ (213961)

19 exp counseling/ (81094)

20 (psychotherap* or psycho-therap*).tw,id. (128685)

21 (behav$ adj3 (intervention$ or program$ or therap$)).tw,id. (77296)

22 ((cognit$ or CBT) adj3 (intervention$ or program$ or therap$)).tw,id. (50383)

23 (psycho$ adj3 (intervention$ or program$ or therap$)).tw,id. (71275)

24 brief therap*.tw,id. (2134)

25 relaxation.tw,id. (16989)

26 mindful$.tw,id. (20356)

27 (counsel?ing or coaching).tw,id. (106343)

28 (third wave adj (psycho$ or therap$)).tw,id. (92)

29 cognit$ restructur$.tw,id. (2673)

30 positive psychology.tw,id. (6013)

31 (refram$ or re-frame$ or reapprais$).tw,id. (11931)

32 (stress adj1 (inoculation or manag$ or reduc$ or resist$)).tw,id. (14666)

33 (anxiety adj3 manage$).tw,id. (2068)

34 "acceptance and commitment ".tw,id. (2919)

35 (multimodal or multi-modal or combined modal$).tw,id. (14938)

36 (health adj3 (educat$ or promot$)).tw,id. (56779)

37 health promotion/ (27059)

38 or/18-37 [Psychotherapy Interventions] (581690)

39 17 or 38 [BA or Psychological Interventions] (648203)

40 comorbidity/ (36863)

41 chronic illness/ (12925)

42 (co-morbid* or comorbid*).ti,ab,id. (67224)

43 (multi-morbid* or multimorbid*).ti,ab,id. (1455)

44 (("long term" or chronic) adj3 (disease? or illness* or condition? or disorder?)).tw,id. (53858)

45 exp diabetes mellitus/ (9532)

46 exp cardiovascular disorders/ (67564)

47 exp lung disorders/ (4961)

48 cerebrovascular accidents/ (22971)

49 exp neoplasms/ (56987)

50 diabet*.tw,id. (34936)

51 (Aneurysm* or Atherosclerosis or Cardio* or endocard* or pericard*).tw,id. (45218)

52 (heart or cardiac or valvular or coronary or arterial or vascular or hypertension).tw,id. (110004)

53 ((long-term or chronic*) adj2 (airway* or pulmonar* or airflow* or lung*) adj2 Obstruct*).tw,id. (2685)

54 (pulmonar* adj2 emphysem*).tw,id. (21)

55 ((long-term or chronic*) adj5 (bronchitis or respirat*)).tw,id. (785)

56 ((long-term or Chronic*) adj2 lung* adj5 (condition* or disease* or symptom* or problem* or failure*)).tw,id. (440)

57 (respiratory adj2 (condition* or disease* or symptom* or problem*)).tw,id. (2445)

58 (stroke or poststroke or post-stroke or cerebrovasc* or brain vasc* or cerebral vasc* or apoplex*).tw,id. (42871)

59 ((brain? or cerebr* or cerebell* or intracran* or intracerebral) adj2 (isch?emi* or infarct* or thrombo* or emboli* or occlus*)).tw,id. (8544)

60 ((brain? or cerebr* or cerebell* or intracerebral or intracranial or subarachnoid) adj2 (h?emorrhage? or h?ematoma* or bleed*)).tw,id. (4719)

61 (transi* adj3 isch?em* adj3 attack?).tw,id. (1261)

62 (cancer* or carcinoma? or tumor? or tumour? or neoplasm? or malignan*).tw,id. (88081)

63 ((noncommunicable or non-communicable or non-infectious or non infectious) adj3 (disease? or illness* or condition? or disorder?)).tw,id. (1378)

64 or/40-63 [NCDs Chronic Disease] (387799)

65 exp major depression/ (147201)

66 (depressi* or depressed).tw,id. (336355)

67 dysthymi*.tw,id. (3994)

68 (mood? or mental health).tw,id. (294087)

69 ((emotion* or psychological) adj (trauma* or distress*)).tw,id. (32561)

70 "common mental disorder*".tw,id. (2287)

71 "depression (emotion)"/ (26517)

72 or/65-71 [Depression] (574810)

73 ((Indian or Indians) not ("west indian*" or "american indian*")).ti,ab,in,id,lo. (19914)

74 india.ti,ab,in,id,lo. (48868)

75 ("south asia*" or bangladesh* or pakistan*).ti,ab,in,id,lo. (12829)

76 (Bhutan* or afghanistan* or maldive* or nepal* or "sri lanka*").ti,ab,in,id,lo. (8367)

77 (Bhutan* or afghanistan* or maldive* or nepal* or "sri lanka*").ti,ab,in,id,lo. (8367)

78 or/73-77 [S Asia] (72746)

79 39 and 64 and 72 and 78 [Psychol Interventions and NCDs and Depression and S Asia] (185)

APA PsycInfo (Ovid) 1806 to April Week 4 2022 2022 Search 2

Date searched: 05/05/2022

Records found: 31

1 ((behavio* adj1 activat*) or BATD).tw,id. (2718)

2 behavio*.mp. and (self adj (evaluat* or monitor*)).tw,id. (6689)

3 (behavio* adj (train* or treatment)).tw,id. (9945)

4 (behavio* adj2 (contracting or modification or modify*)).tw,id. (8564)

5 reinforc*.ti,id. (28214)

6 (reinforce or reinforcer or reinforcement or reinforcements or re-inforcement or re-inforcements).ab. /freq=2 (16892)

7 (reinforc* adj3 (behavio* or environment* or experience*)).tw,id. (7292)

8 (reinforc* adj1 (positive or contingent)).tw,id. (4889)

9 (activit* adj2 schedul*).tw,id. (659)

10 ((pleas* or enjoy* or reward*) adj4 (activit* or event?)).tw,id. (4925)

11 ((operant or instrumental) adj (conditioning or learning)).tw,id. (6306)

12 (positive interaction* or avoida* coping or environmental contingenc* or contingency management).tw,id. (6389)

13 functional analysis.tw,id. (3476)

14 ((gain? or reapprais*) adj2 focus*).tw,id. (154)

15 ((psychoeducat* or psycho-educat*) and (behavi* or coping or self manag*)).ti,ab,id. (5599)

16 behavioral activation system/ (569)

17 or/1-16 [Behavioural Activation] (87581)

18 exp psychotherapy/ (213961)

19 exp counseling/ (81094)

20 (psychotherap* or psycho-therap*).tw,id. (128685)

21 (behav$ adj3 (intervention$ or program$ or therap$)).tw,id. (77296)

22 ((cognit$ or CBT) adj3 (intervention$ or program$ or therap$)).tw,id. (50383)

23 (psycho$ adj3 (intervention$ or program$ or therap$)).tw,id. (71275)

24 brief therap*.tw,id. (2134)

25 relaxation.tw,id. (16989)

26 mindful$.tw,id. (20356)

27 (counsel?ing or coaching).tw,id. (106343)

28 (third wave adj (psycho$ or therap$)).tw,id. (92)

29 cognit$ restructur$.tw,id. (2673)

30 positive psychology.tw,id. (6013)

31 (refram$ or re-frame$ or reapprais$).tw,id. (11931)

32 (stress adj1 (inoculation or manag$ or reduc$ or resist$)).tw,id. (14666)

33 (anxiety adj3 manage$).tw,id. (2068)

34 "acceptance and commitment ".tw,id. (2919)

35 (multimodal or multi-modal or combined modal$).tw,id. (14938)

36 (health adj3 (educat$ or promot$)).tw,id. (56779)

37 health promotion/ (27059)

38 or/18-37 [Psychotherapy Interventions] (581690)

39 17 or 38 [BA or Psychological Interventions] (648203)

40 comorbidity/ (36863)

41 chronic illness/ (12925)

42 (co-morbid* or comorbid*).ti,ab,id. (67224)

43 (multi-morbid* or multimorbid*).ti,ab,id. (1455)

44 (("long term" or chronic) adj3 (disease? or illness* or condition? or disorder?)).tw,id. (53858)

45 exp diabetes mellitus/ (9532)

46 exp cardiovascular disorders/ (67564)

47 exp lung disorders/ (4961)

48 cerebrovascular accidents/ (22971)

49 exp neoplasms/ (56987)

50 diabet*.tw,id. (34936)

51 (Aneurysm* or Atherosclerosis or Cardio* or endocard* or pericard*).tw,id. (45218)

52 (heart or cardiac or valvular or coronary or arterial or vascular or hypertension).tw,id. (110004)

53 ((long-term or chronic*) adj2 (airway* or pulmonar* or airflow* or lung*) adj2 Obstruct*).tw,id. (2685)

54 (pulmonar* adj2 emphysem*).tw,id. (21)

55 ((long-term or chronic*) adj5 (bronchitis or respirat*)).tw,id. (785)

56 ((long-term or Chronic*) adj2 lung* adj5 (condition* or disease* or symptom* or problem* or failure*)).tw,id. (440)

57 (respiratory adj2 (condition* or disease* or symptom* or problem*)).tw,id. (2445)

58 (stroke or poststroke or post-stroke or cerebrovasc* or brain vasc* or cerebral vasc* or apoplex*).tw,id. (42871)

59 ((brain? or cerebr* or cerebell* or intracran* or intracerebral) adj2 (isch?emi* or infarct* or thrombo* or emboli* or occlus*)).tw,id. (8544)

60 ((brain? or cerebr* or cerebell* or intracerebral or intracranial or subarachnoid) adj2 (h?emorrhage? or h?ematoma* or bleed*)).tw,id. (4719)

61 (transi* adj3 isch?em* adj3 attack?).tw,id. (1261)

62 (cancer* or carcinoma? or tumor? or tumour? or neoplasm? or malignan*).tw,id. (88081)

63 ((noncommunicable or non-communicable or non-infectious or non infectious) adj3 (disease? or illness* or condition? or disorder?)).tw,id. (1378)

64 or/40-63 [NCDs Chronic Disease] (387799)

65 exp mental disorders/ (929930)

66 exp Behavior Disorders/ (61735)

67 (mental or mentally or psychiatr* or psycho* or depressi* or depressed or MDD or anxi* or phobia or phobic or agoraphobi* or dysthymi* or ADNOS).tw,id. (1809039)

68 (schizo* or hebephrenic* or oligophreni* or akathisi* or acathisi* or neuroleptic-induc*).tw,id. (139015)

69 (somatoform or somatiz* or somatis* or hysteri* or briquet or multisomat* or multi somat* or MUPs or medically unexplained).tw,id. (17469)

70 ((dissociative adj3 (disorder* or reaction*)) or dissociation).tw,id. (21709)

71 (affective* adj (disorder? or disease? or illness* or symptom?)).tw,id. (21644)

72 (PTSD or psychological trauma or psychotrauma* or combat disorder? or war disorder?).tw,id. (40346)

73 ((post-trauma* or posttrauma*) adj3 (stress* or disorder?)).tw,id. (47723)

74 ((stress or cognitive or cognition or personality or impulse control or mood or paranoid or psychotic or neurologic* or nervous or nervous system or eating) adj (disorder? or illness* or disease?)).tw,id. (151372)

75 ((bipolar or behavio?ral or obsessive or compulsive or panic or mood or delusional) adj (disorder? or illness* or disease?)).tw,id. (79810)

76 (trichotillomani* or OCD or obsess*-compulsi* or GAD or stress reaction? or acute stress or neuros#s or neurotic).tw,id. (63368)

77 (stress syndrome? or distress syndrome? or pain disorder? or dementia or alzheimer? or epilepsy).tw,id. (149409)

78 ((substance abuse or "substance use" or drug abuse or "drug use") adj2 disorder?).tw,id. (22814)

79 (personality adj2 disorder?).tw,id. (36534)

80 (sleep? adj2 (disorder? or syndrome?)).tw,id. (10927)

81 or/65-80 [Mental Disorders] (2253817)

82 (bangladesh* or pakistan*).ti,ab,in,id,lo. (10354)

83 39 and 64 and 81 and 82 [Psy Interventions NCDs Mental Disorders_Pakistan Bangladesh] (31)

APA PsycInfo (Ovid) 1806 to April Week 4 2022 2022 Search 1

Date searched: 05/05/2022

Records found: 153

1 ((behavio* adj1 activat*) or BATD).tw,id. (2718)

2 behavio*.mp. and (self adj (evaluat* or monitor*)).tw,id. (6689)

3 (behavio* adj (train* or treatment)).tw,id. (9945)

4 (behavio* adj2 (contracting or modification or modify*)).tw,id. (8564)

5 reinforc*.ti,id. (28214)

6 (reinforce or reinforcer or reinforcement or reinforcements or re-inforcement or re-inforcements).ab. /freq=2 (16892)

7 (reinforc* adj3 (behavio* or environment* or experience*)).tw,id. (7292)

8 (reinforc* adj1 (positive or contingent)).tw,id. (4889)

9 (activit* adj2 schedul*).tw,id. (659)

10 ((pleas* or enjoy* or reward*) adj4 (activit* or event?)).tw,id. (4925)

11 ((operant or instrumental) adj (conditioning or learning)).tw,id. (6306)

12 (positive interaction* or avoida* coping or environmental contingenc* or contingency management).tw,id. (6389)

13 functional analysis.tw,id. (3476)

14 ((gain? or reapprais*) adj2 focus*).tw,id. (154)

15 ((psychoeducat* or psycho-educat*) and (behavi* or coping or self manag*)).ti,ab,id. (5599)

16 behavioral activation system/ (569)

17 or/1-16 [Behavioural Activation] (87581)

18 exp psychotherapy/ (213961)

19 exp counseling/ (81094)

20 (psychotherap* or psycho-therap*).tw,id. (128685)

21 (behav$ adj3 (intervention$ or program$ or therap$)).tw,id. (77296)

22 ((cognit$ or CBT) adj3 (intervention$ or program$ or therap$)).tw,id. (50383)

23 (psycho$ adj3 (intervention$ or program$ or therap$)).tw,id. (71275)

24 brief therap*.tw,id. (2134)

25 relaxation.tw,id. (16989)

26 mindful$.tw,id. (20356)

27 (counsel?ing or coaching).tw,id. (106343)

28 (third wave adj (psycho$ or therap$)).tw,id. (92)

29 cognit$ restructur$.tw,id. (2673)

30 positive psychology.tw,id. (6013)

31 (refram$ or re-frame$ or reapprais$).tw,id. (11931)

32 (stress adj1 (inoculation or manag$ or reduc$ or resist$)).tw,id. (14666)

33 (anxiety adj3 manage$).tw,id. (2068)

34 "acceptance and commitment ".tw,id. (2919)

35 (multimodal or multi-modal or combined modal$).tw,id. (14938)

36 (health adj3 (educat$ or promot$)).tw,id. (56779)

37 health promotion/ (27059)

38 or/18-37 [Psychotherapy Interventions] (581690)

39 17 or 38 [BA or Psychological Interventions] (648203)

40 comorbidity/ (36863)

41 chronic illness/ (12925)

42 (co-morbid* or comorbid*).ti,ab,id. (67224)

43 (multi-morbid* or multimorbid*).ti,ab,id. (1455)

44 (("long term" or chronic) adj3 (disease? or illness* or condition? or disorder?)).tw,id. (53858)

45 exp diabetes mellitus/ (9532)

46 exp cardiovascular disorders/ (67564)

47 exp lung disorders/ (4961)

48 cerebrovascular accidents/ (22971)

49 exp neoplasms/ (56987)

50 diabet*.tw,id. (34936)

51 (Aneurysm* or Atherosclerosis or Cardio* or endocard* or pericard*).tw,id. (45218)

52 (heart or cardiac or valvular or coronary or arterial or vascular or hypertension).tw,id. (110004)

53 ((long-term or chronic*) adj2 (airway* or pulmonar* or airflow* or lung*) adj2 Obstruct*).tw,id. (2685)

54 (pulmonar* adj2 emphysem*).tw,id. (21)

55 ((long-term or chronic*) adj5 (bronchitis or respirat*)).tw,id. (785)

56 ((long-term or Chronic*) adj2 lung* adj5 (condition* or disease* or symptom* or problem* or failure*)).tw,id. (440)

57 (respiratory adj2 (condition* or disease* or symptom* or problem*)).tw,id. (2445)

58 (stroke or poststroke or post-stroke or cerebrovasc* or brain vasc* or cerebral vasc* or apoplex*).tw,id. (42871)

59 ((brain? or cerebr* or cerebell* or intracran* or intracerebral) adj2 (isch?emi* or infarct* or thrombo* or emboli* or occlus*)).tw,id. (8544)

60 ((brain? or cerebr* or cerebell* or intracerebral or intracranial or subarachnoid) adj2 (h?emorrhage? or h?ematoma* or bleed*)).tw,id. (4719)

61 (transi* adj3 isch?em* adj3 attack?).tw,id. (1261)

62 (cancer* or carcinoma? or tumor? or tumour? or neoplasm? or malignan*).tw,id. (88081)

63 ((noncommunicable or non-communicable or non-infectious or non infectious) adj3 (disease? or illness* or condition? or disorder?)).tw,id. (1378)

64 or/40-63 [NCDs Chronic Disease] (387799)

65 exp major depression/ (147201)

66 (depressi* or depressed).tw,id. (336355)

67 dysthymi*.tw,id. (3994)

68 (mood? or mental health).tw,id. (294087)

69 ((emotion* or psychological) adj (trauma* or distress*)).tw,id. (32561)

70 "common mental disorder*".tw,id. (2287)

71 "depression (emotion)"/ (26517)

72 or/65-71 [Depression] (574810)

73 ((Indian or Indians) not ("west indian*" or "american indian*")).ti,ab,in,id,lo. (19914)

74 india.ti,ab,in,id,lo. (48868)

75 ("south asia*" or bangladesh* or pakistan*).ti,ab,in,id,lo. (12829)

76 (Bhutan* or afghanistan* or maldive* or nepal* or "sri lanka*").ti,ab,in,id,lo. (8367)

77 (Bhutan* or afghanistan* or maldive* or nepal* or "sri lanka*").ti,ab,in,id,lo. (8367)

78 or/73-77 [S Asia] (72746)

79 39 and 64 and 72 and 78 [Psychol Interventions and NCDs and Depression and S Asia] (185)

WPRO Western Pacific Region Index Medicus (WHO Global Health Index Medicus) Search 1

Date searched: 05/05/2022

Records found: 11

Same search strategy as IMEAR

WPRO Western Pacific Region Index Medicus (WHO Global Health Index Medicus) Search 2

Date searched: 05/05/2022

Records found: 1

Same search strategy as IMEAR

**Search Methods**

Updated 05-05-2022

### Methods

In October 2020 (16^th^ Oct – 19^th^ Oct) and on 5^th^ May 2022 we searched the following databases for studies of behavioural activation or psychological interventions used for addressing depression in people with NCDs in Bangladesh and Pakistan.

· Applied Social Sciences Index and Abstracts (ASSIA)‎ (ProQuest) 1987+

· CINAHL (EBSCOhost) 1981+

· Embase Classic+Embase (Ovid) 1947 to 2022 May 04

· Global Health (Ovid) 1910 to 2022 Week 17

· IMEMR Index Medicus for the Eastern Mediterranean (WHO Global Health Index Medicus).

· ISMEAR Index Medicus for the South East Asia Region (WHO Global Health Index Medicus)

· Ovid MEDLINE(R) ALL 1946 to May 04, 2022

· APA PsycInfo (Ovid) 1806 to April Week 4 2022

· WPRO Western Pacific Region Index Medicus (WHO Global Health Index Medicus)

We devised two search strategies to be run in each database; a primary search of S Asian literature reporting depression, supplemented by a second search focussed on Bangladesh/Pakistan studies reporting any mental health disorder.

Searches were developed for the concepts:

· Search 1: Behavioural Activation/Psychological interventions AND NCDs AND Depression AND S Asia

· Search 2 : Behavioural Activation /Psychological interventions AND NCDs AND Mental Disorders AND Bangladesh/Pakistan

The searches were developed iteratively, starting with previously developed search strategies for behavioural activation (UPHOFF), Psychological Interventions (KUNZLER), NCDs (UPHOFF), mental disorders (MISHU), and refining strategies iteratively to address the two search questions. Subject headings and free text words were used in each search concept. Language and publication date limits were not used. Searches were designed by the Information Specialist and agreed with project team members. The search was peer-reviewed by a second Information Specialist using the PRESS checklist [McGowan ref]. Please see Appendix [X] for full search strategies.

The results of the database searches were stored and de-duplicated in an EndNote library [ADD if personal library references and citation tracking refs were also stored in EndNote].

Further relevant studies were sought by citation searching (forwards and backwards) of the included studies, and hand searches of [LIST SOURCE] [IF APPLICABLE]

### Results

The database searches identified 2823 records. Once duplicates were removed there were 1771 records. Citation searches identified [NUMBER] records. Handsearching identified [NUMBER] records.

*Breakdown per database for PRISMA diagram:*

ASSIA n=158

CINAHL n=334

Embase n=1215

Global Health n=221

IMEMR n=21

ISMEAR n=19

MEDLINE n=627

PsycInfo n=216

WPRO n=12

**REFS:**

Kunzler AM, Helmreich I, König J, Chmitorz A, Wessa M, Binder H, Lieb K. Psychological interventions to foster resilience in healthcare students. Cochrane Database of Systematic Reviews 2020, Issue 7. Art. No.: CD013684. DOI: 10.1002/14651858.CD013684

Uphoff E, Pires M, Barbui C, Barua D, Churchill R, Cristofalo D, Ekers D, Fottrell E, Mazumdar P, Purgato M, Rana R, Wright J, Siddiqi N. Behavioural activation therapy for depression in adults with non‐communicable diseases. Cochrane Database of Systematic Reviews 2020, Issue 8. Art. No.: CD013461. DOI: 10.1002/14651858.CD013461.pub2.

Mishu MP, Peckham EJ, Wright J, Taylor J, Tirbhowan N, Ajjan R, Al Azdi Z, Stubbs B, Churchill R, Siddiqi N. Interventions for preventing type 2 diabetes in adults with mental disorders in low and middle income countries. Cochrane Database of Systematic Reviews 2019, Issue 3. Art. No.: CD013281. DOI: 10.1002/14651858.CD013281.

McGowan J, Sampson M, Salzwedel DM, Cogo E, Foerster V, Lefebvre C. PRESS peer review of electronic search strategies: 2015 guideline statement. Journal of clinical epidemiology. 2016 Jul 1;75
