# Supplementary material for: Effectiveness and implementation of psychological interventions for depression in people with non-communicable diseases in South Asia: Systematic review and meta-analysis
Source: Int J Ment Health. 2023 Apr 24;52(3):260–84. doi: 10.1080/00207411.2023.2202431 (PMC10461698; doi:10.1080/00207411.2023.2202431)
Supplement: Supplemental Material [file MIMH_A_2202431_SM3375.zip › Appendix 2.docx]

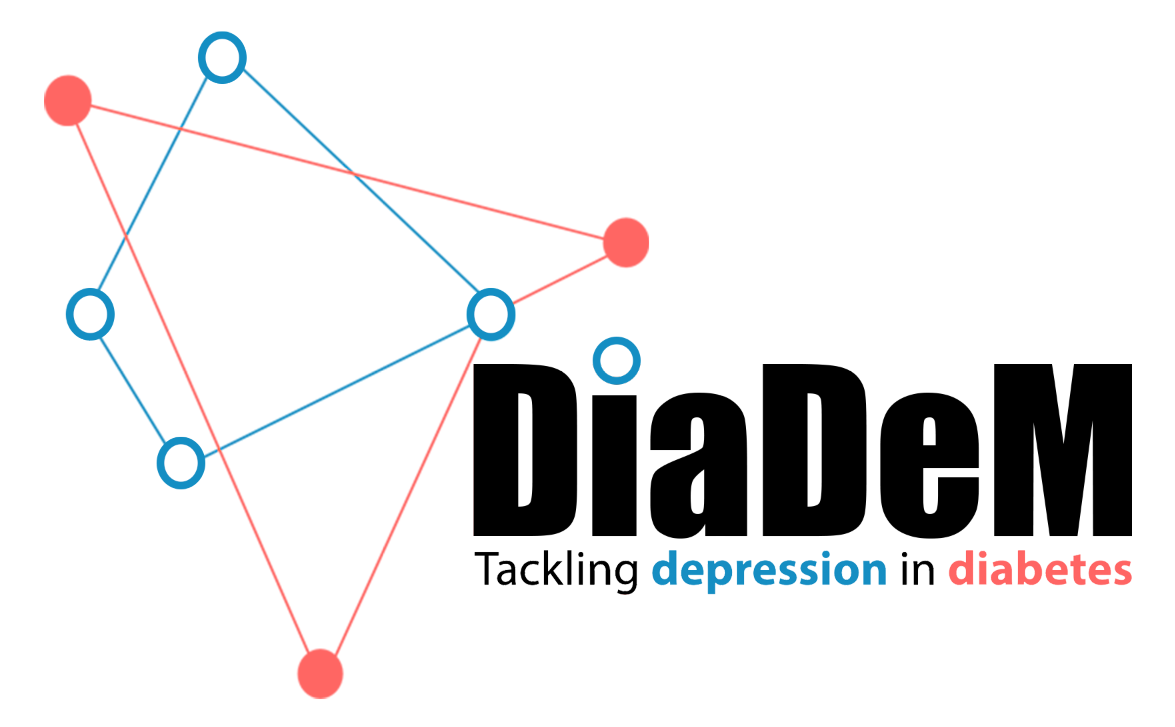


#

#

**Psychological interventions for depression in people with non-communicable diseases in South Asia**

**Appendix 2. List of policy documents**

Supplementary table 1. Policy documents

| No | Name of document | Country | Type of document |
| --- | --- | --- | --- |
| 1 | 5 Year Health Plan | Pakistan | Policy |
| 2 | National Health Vision 2016-2025 | Pakistan | Strategic Vision |
| 3 | Non-Communicable Diseases & Mental Health National Action Framework 2021-30 | Pakistan | Policy |
| 3 | Islamabad Capital Territory Health Strategy 2019-2023 | Pakistan | Strategy |
| 4 | Health Policy Khyber Pakhtunkhwa (KP) 2018 | Pakistan | Policy |
| 5 | Khyber Pakhtunkhwa Health Sector Strategic Plan 2019 – 2025 (and associated implementation plan) | Pakistan | Strategic Plan |
| 6 | Secondary Level Minimum Health Services Delivery Package for Secondary Care KP 2016 | Pakistan | Policy |
| 7 | Khyber Pakhtunkhwa Public Health Surveillance and Response Act 2017 | Pakistan | Legislation |
| 8 | Khyber Pakhtunkhwa Mental Health Act 2017 | Pakistan | Legislation |
| 9 | Sindh Health Sector Strategy 2012-2020 | Pakistan | Policy |
| 10 | Sindh Mental Health Act (+Sindh Mental Health Rules 2014) 2013 | Pakistan | Legislation |
| 11 | Punjab Provincial Strategic Plan (2016 – 2020) | Pakistan | Policy |
| 12 | The Punjab Healthcare Commission Act 2010 | Pakistan | Legislation |
| 13 | Balochistan Health Policy 2018-2025 | Pakistan | Policy |
| 14 | Balochistan Health Strategy 2018-2025 | Pakistan | Strategy |
| 15 | Integration of MH into Primary Care, 2018 | Pakistan | Research article |
| 16 | Pakistan at a Glance 2018 | Pakistan | WHO summary |
| 17 | Stakeholders’ perspective on mental health laws in Pakistan, 2020 | Pakistan | Research article |
| 18 | Non-communicable diseases in Pakistan: a health system perspective, 2018 | Pakistan | Journal article |
| 19 | Current management strategies to target the increasing diabetes within Pakistan, 2018 | Pakistan | Journal article |
| 20 | Multisectoral action plan for the prevention and control of NCDs 2018-2025 (with associated 3 year operational plan) | Bangladesh | Strategic plan |
| 21 | Health Nutrition and Population Strategic Investment Plan (HNPSIP) 2018-2021 | Bangladesh | Strategic plan |
| 22 | 4^th^ Health, Population and Nutrition Sector Program (2017-2022), mid-term review 2020 | Bangladesh | Programme review |
| 23 | National Mental Health Act (Bangla) | Bangladesh | Legislation |
| 24 | Hypertension and Type-2 Diabetes in Bangladesh : Continuum of Care Assessment and Opportunities for Action (2019) | Bangladesh | World Bank Report |
| 25 | National drug policy reform for non-communicable diseases in low-income countries: an example from Bangladesh | Bangladesh | WHO report |
| 26 | Bangladesh WHO Special Initiative for Mental Health Situational Assessment (2020) | Bangladesh | WHO report |
| 27 | Bangladesh at a glance 2018 | Bangladesh | WH0 summary |
| 28 | 2019 Health Bulletin | Bangladesh | Government summary |
| 29 | Integration of mental health with primary care in Bangladesh | Bangladesh | Government report? |
| 30 | Mental Health rules (Bangla) | Bangladesh | Policy |
| 31 | The new mental health act. Lancet commentary | Bangladesh | Journal commentary |
| 32 | The new mental health act. Lancet commentary - response | Bangladesh | Journal commentary |
| 33 | Mental health Act: England perspective and the scenario of Bangladesh | Bangladesh | Workshop report |
| 34 | Integrating Mental health into primary care | Bangladesh | Journal commentary |
